# Obesity and Risk for Brain/CNS Tumors, Gliomas and Meningiomas: A Meta-Analysis

**DOI:** 10.1371/journal.pone.0136974

**Published:** 2015-09-02

**Authors:** Theodoros N. Sergentanis, Georgios Tsivgoulis, Christina Perlepe, Ioannis Ntanasis-Stathopoulos, Ioannis-Georgios Tzanninis, Ioannis N. Sergentanis, Theodora Psaltopoulou

**Affiliations:** 1 Department of Hygiene, Epidemiology and Medical Statistics, Medical School, National University of Athens, Athens, Greece; 2 Second Department of Neurology, “Attikon” University Hospital, Medical School, National University of Athens, Athens, Greece; 3 Hôpital de Psychiatrie, Hôpitaux Universitaires de Genève, Geneva, Switzerland; University of Alabama at Birmingham, UNITED STATES

## Abstract

**Objective:**

This meta-analysis aims to examine the association between being overweight/obese and risk of meningiomas and gliomas as well as overall brain/central nervous system (CNS) tumors.

**Study Design:**

Potentially eligible publications were sought in PubMed up to June 30, 2014. Random-effects meta-analysis and dose-response meta-regression analysis was conducted. Cochran Q statistic, I-squared and tau-squared were used for the assessment of between-study heterogeneity. The analysis was performed using Stata/SE version 13 statistical software.

**Results:**

A total of 22 studies were eligible, namely 14 cohort studies (10,219 incident brain/CNS tumor cases, 1,319 meningioma and 2,418 glioma cases in a total cohort size of 10,143,803 subjects) and eight case-control studies (1,009 brain/CNS cases, 1,977 meningioma cases, 1,265 glioma cases and 8,316 controls). In females, overweight status/obesity was associated with increased risk for overall brain/CNS tumors (pooled RR = 1.12, 95%CI: 1.03–1.21, 10 study arms), meningiomas (pooled RR = 1.27, 95%CI: 1.13–1.43, 16 study arms) and gliomas (pooled RR = 1.17, 95%CI: 1.03–1.32, six arms). Obese (BMI>30 kg/m^2^) females seemed particularly aggravated in terms of brain/CNS tumor (pooled RR = 1.19, 95%CI: 1.05–1.36, six study arms) and meningioma risk (pooled RR = 1.48, 95%CI: 1.28–1.71, seven arms). In males, overweight/obesity status correlated with increased meningioma risk (pooled RR = 1.58, 95%CI: 1.22–2.04, nine study arms), whereas the respective association with overall brain/CNS tumor or glioma risk was not statistically significant. Dose-response meta-regression analysis further validated the findings.

**Conclusion:**

Our findings highlight obesity as a risk factor for overall brain/CNS tumors, meningiomas and gliomas among females, as well as for meningiomas among males.

## Introduction

Glioma and meningioma are the two most common primary central nervous system (CNS) tumors, representing 70% and 20% of brain tumors, respectively [1, 2]. Gliomas originate from glial cells, are as a rule histologically malignant and are more frequent among males [1]. On the other hand, meningiomas originate from the arachnoidal cells of the leptomeninges, are typically histologically benign and are two-fold more frequent among females [3]. The risk factors for brain tumors are poorly understood [4], but may include genetic conditions and ionizing radiation [5]; occupational exposures seem also to be meaningful, as glioma has been linked with occupational exposure to arsenic, mercury and petroleum products, whereas meningioma has been associated with lead exposure [6]. The Million Women Study highlighted attained height as a risk factor for the incidence of all central nervous system tumors with an excess risk of about 20% per 10 cm increase in height [7]. Currently, there is a vivid debate regarding the effects of mobile phone use [8]. On the other hand, a meta-analysis has highlighted atopy as a potential factor inversely associated with glioma but not meningioma [9], whereas another meta-analysis did not find any significant association between smoking and glioma risk [10]. Head injury may entail at most only a small increase in the overall risk of brain tumors[11]. Hormonal and reproductive risk factors appear to be associated with meningioma risk, as positive associations with hormone replacement therapy use [12], uterine fibroids [13] and endometriosis [13] have been reported, culminating at a possibly positive association with breast cancer [14].

Obesity is a well established risk factor for several cancer types [15]; it has been postulated that obesity may account for approximately 20% of all cancer cases [16]. The spectrum of obesity-related cancer may span colon [17], endometrial [18], postmenopausal breast [16], renal [15, 16], esophageal [15], thyroid [15, 16], prostate [19] cancer and hematological malignancies [20], whereas a recent meta-analysis performed by our team highlighted the association between obesity and melanoma among males [21]. Nevertheless, the association between obesity and CNS tumors remains rather obscure, given that CNS tumors are rather uncommon and individual studies have yielded mutually conflicting results. Recently, a meta-analysis focused especially on meningioma, synthesizing data from six studies [22]; the authors concluded that being obese, but not overweight, was associated with increased risk for meningioma, especially among females.

To our knowledge, however, no effort has been undertaken till now, to quantitatively synthesize all published cohort and case-control studies, in order to evaluate the potential association between glioma, or overall brain/CNS tumors and obesity.

In view of the former considerations, our aim was to comprehensively examine the association between obesity and risk for brain/CNS tumors, glioma as well as meningioma in adults, synthesizing all available evidence. Separate analyses were conducted in males and females, in order to evaluate potential sex-specific discrepancies.

## Materials and Methods

### Search algorithm and eligibility of studies

This meta-analysis was performed in line with the PRISMA guidelines. Eligible studies were identified in PubMed; no restriction regarding publication language was adopted and the end-of-search date was June 30, 2014. The details about the search algorithm are provided in S1 File.

Cohort and case-control studies that examined the association between BMI and risk of overall brain/CNS tumors, glioma or meninigioma were deemed eligible. A systematic search in the reference lists of eligible articles and relevant reviews for potentially eligible articles was performed (“snowball” procedure). In case of overlapping study populations the larger study was ultimately included. Two reviewers (TNS, TP) independently performed the selection of studies; in case of disagreement, the final decision was reached by team consensus.

### Data extraction

The data extraction from eligible studies included: first author’s name, publication year, journal where the study was published, type of study (cohort or case-control), study period including the follow-up, geographical region, age of subjects, adjustment factors in multivariable analyses. Specifically for case-control studies, the number, definition and eligibility criteria of cases and controls, as well as the matching factors were extracted. Regarding cohort studies, the cohort size and number of incident cases were abstracted. Two reviewers (IN-S, IGT) independently extracted the data and, in case of disagreement, final decision was reached by team consensus.

Regarding effect estimates, the maximally adjusted Odds Ratios (ORs) for case-control studies as well as the maximally adjusted Relative Risks (RRs) for cohort studies were abstracted, together with their Confidence Intervals (CIs) for each BMI category. Separate abstraction was undertaken for males and females. When the aforementioned information was not available, the data of 2x2 tables in the articles were used for the calculation of crude effect estimates and 95% CIs.

Letters were sent to the authors of studies which did not separately report relative risks pertaining to overweight and obese subjects, as well as to articles reporting exclusively on overall brain/CNS cancer, requesting the subgroup analyses on gliomas and meningiomas. The corresponding authors were contacted twice (a reminder e-mail was sent one week after the first e-mail).

### Statistical analyses: meta-analysis

Being overweight was defined as BMI 25–30 kg/m^2^ and obesity was defined as BMI >30 kg/m^2^. In this analysis, the term “study arms” refers to the various BMI categories presented in the included studies. The overweight and/or obese “study arms” were pooled, as appropriate. Apart from the overall pooling of overweight/obese subjects (vs. normal weight), subgroup analyses were performed for overweight and obese subjects. Separate models were constructed regarding overall brain/CNS tumors, meningiomas and gliomas, stratified by gender (females; males; study arms not distinguishing between the two sexes). The pooled effect estimate was calculated on the basis of random effects models (DerSimonian-Laird, with the estimate of heterogeneity being taken from the Mantel-Haenszel model). Cochran *Q* statistic and *I* ^*2*^ were used for the assessment of between-study heterogeneity [23]; nevertheless, in view of the limitations of I^2^ as an index [24, 25] the values of τ^2^ (tau^2) were also provided.

### Statistical analyses: dose-response meta-regression analysis

Dose-response meta-regression analysis serves the investigation of the relationship between different BMI levels and the outcomes. A specific BMI value was allocated to each study arm according to the algorithm described by our previous meta-analysis [21]. Specifically, in case of category *a* to *b* the arithmetic mean was adopted. Regarding the upper, open ended categories (≥*a*), two alternative approaches were undertaken [21], namely: (i) the lower bound was multiplied by 1.2 (approach according to Berlin et al. [26]) and (ii) the formula *a*_*n*_+ (*a*_*n*_ *—a*_*n-1*_) was implemented (approach according to Il’yasova et al. [27]), where *a*_*n*_ stands for the open-ended category threshold (>*a*_*n*_) and *a*_*n-1*_ pertains to the immediately lower category (*a*_*n-1*_ *to a*_*n*_). During pooling of study arms, the values according to the approach by Berlin et al. [26] were preferred, because this algorithm allocates values of upper bounds to a greater number of study arms versus that by Il’yasova et al. [27]. As described in our previous meta-analysis [21], the latter algorithm cannot allocate values when the *n-1* category is open, as is the case in studies adopting a binary categorization.

At the meta-regression analysis, the log of the study effect estimates was set as the dependent variable in a general linear model. BMI was analyzed in increments of 5 kg/m^2^ [21]; we present the exponentiated slope coefficient from the linear regression. Both alternative analyses (approaches by Berlin et al. [26] and Il’yasova et al. [27]) were presented. All analyses were performed using Stata/SE version 13 (Stata Corp, College Station, TX, USA).

### Risk of bias

The Newcastle-Ottawa Quality scale [28] was used for the evaluation of quality regarding the included studies. With respect to longitudinal cohort studies, the cut-off value was *a priori* set at 5 years regarding the desirable length of follow-up, whereas the cut-off value for completeness of follow-up was set at 85%. The studies were rated by two independent reviewers (IN-S and IGT); in case of disagreements, final decision was reached by team consensus.

Concerning the assessment of publication bias, the overall (overweight and obese pooled together) pooling analyses in males and females were chosen, in order to maximize the power of the relevant tests [23]. The Egger’s [29] formal statistical test was adopted; for the interpretation of Egger’s test, statistical significance was defined as p<0.1.

## Results

### Selection and description of eligible studies

**Fig 1:**
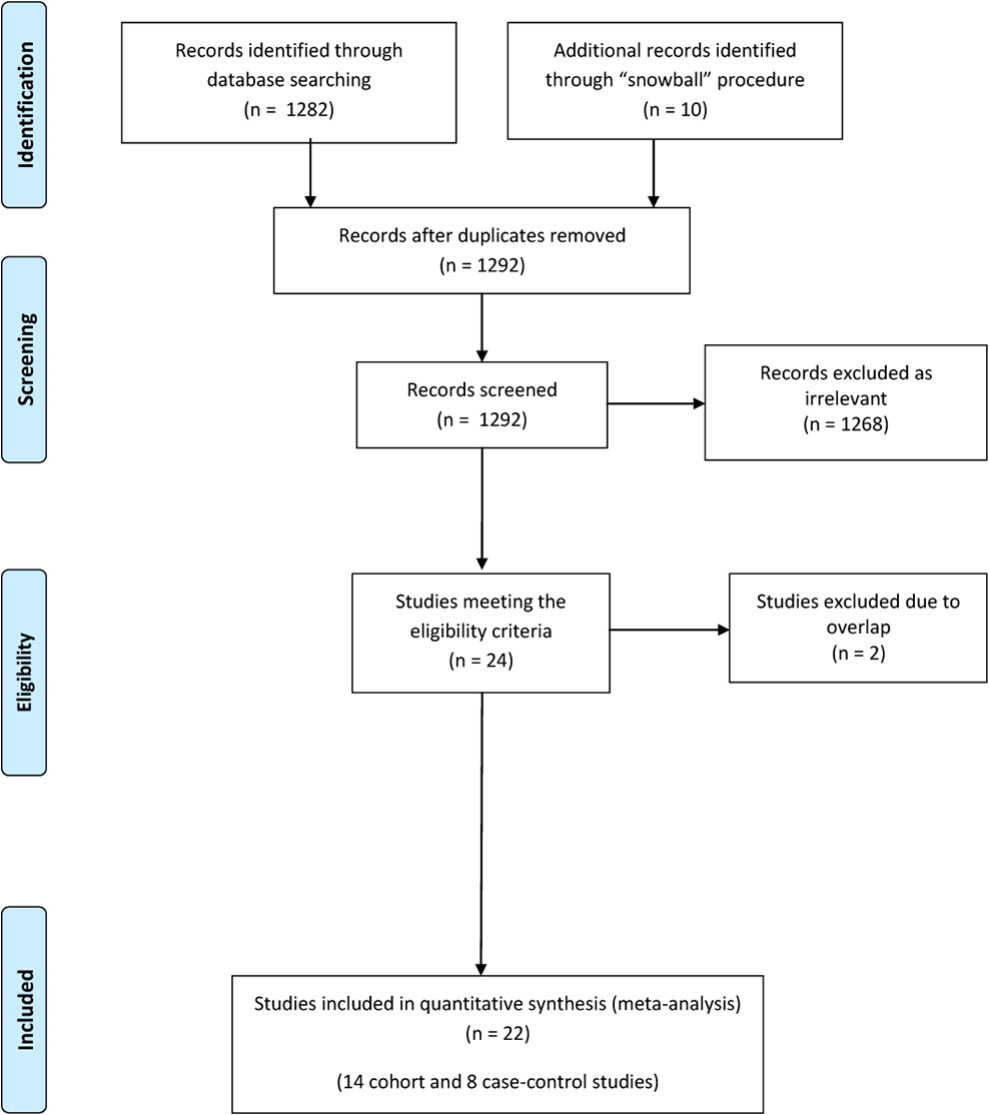
Flow chart describing the successive steps during the selection of eligible studies.

Fig 1 presents the flow chart describing the successive steps during the selection of eligible studies. A total of 1,282 abstracts were identified and screened; all details regarding the selection of eligible studies are presented in S1 File.

Taken as a whole, 14 cohort studies [7, 30–42] including 10,219 incident brain/CNS tumor cases [7, 30, 31, 37–40, 42], 1,319 incident meningioma cases [7, 31, 33–35, 41], 2,418 incident glioma cases [7, 31, 32, 35, 36, 41], in a total cohort size of 10,143,803 subjects (Table 1) and eight case-control studies [43–50] including 1,009 brain/CNS cases, 1,977 meningioma cases, 1,265 glioma cases and 8,316 controls (Table 2) were included in the present meta-analysis. The dataset of the study is available in “S1 Dataset” and the PRISMA Checklist in “S1 Checklist”.

**Table 1:** Characteristics of the included cohort studies.

Study (year)	Cohort size	Brain/CNS cancer cases	Meningioma cases	Glioma cases	Follow-up (years, median or mean)	Study period	Region	Age range	Cohort Characteristics	Definition of glioma, meningioma and brain/CNS tumors in cohort	Type of ascertainment of BMI	Adjusting factors
Wolk (2001)	28,129	66 (brain)	0	0	10.3	1965–31 December 1993	Sweden	> = 18	All patients recorded in the Inpatient Register of the National Board of Health and Wellfare with a discharge diagnosis of obesity were initially included, rendering a total of 36,159 unique IDs. 4,799 records were excluded from the cohort due to erroneous or incomplete national registration numbers or inconsistencies discovered during record linkage. Another 1,013 patients with prevalent cancer and 200 patients with cancers that occurred in the first year of follow up were excluded to minimize the possible selection and detection biases. 127 cancer cases diagnosed after death were also excluded due to differences in autopsy rates between hospitalized patients with obesity and the general population.	Diagnosis stated in the Inpatient Register of the National Board of Health and Wellfare.	Diagnosis stated in the Inpatient Register of the National Board of Health and Wellfare. Men were classified as obese when their BMI was higher than 30 kg/m^2^ whereas women were classified as obese when their BMI was higher than 28.6 kg/m^2^.	Age, calendar year (inherent adjustments in SIR-Standardized Incidence Ratio))
Calle (2003)	900,053	1655 (brain)	0	0	16	1982–31 December 1998	USA, District of Columbia, Puerto Rico	> = 30	Families that had at least one member aged 45 years or older, from the participants of the Cancer Prevention Study II. Participants completed a confidential, mailed questionnaire in 1982. Participants whose values for height and weight in the questionnaire were missing, whose weight one year before the interview was unknown or who had lost more than 4.5 kg that last year were excluded from the study. Also participants with a below normal weight according to WHO guidelines, cancer (other than non-melanoma skin cancer) at base line and those missing information on race or smoking history were also excluded.	Fatal cases; for deaths occurring before September 1988 information were ascertained by means of personal inquiries made by volunteers in September 1984, 1986 and 1988. The deaths occurring after September 1988 (93% of all deaths) were ascertained by linkage with the National Death Index.	Information from confidential mailed questionnaire.	Age, education, smoking status, and number of cigarettes smoked, physical activity, alcohol use, marital status, race, aspirin use, estrogen replacement therapy (in women), fat consumption and vegetable consumption
Jhawar (2003)	121700	0	125	0	9.97	1976–1996	11 US states enrolled in the NHS (Nurses Health Study) cohort	30–55	Nurses’ Health Study cohort; all participants were women nurses who completed baseline questionnaires regarding risk factors for cancer, cardiovascular disease, and a variety of other health conditions. Since enrollment, these women have been followed up by means of biennial mailed questionnaires, updating exposure information and onset of newly diagnosed disease. At each follow-up interval all women who reported having any cancer other than nonmelanoma skin cancer were excluded from subsequent analyses.	Self-reported diagnosis of meningioma on the supplemental questionnaire or medical record	Self reported, updated at each follow-up interval, height and weight information was used to calculate and re-calculate the BMI	Age, menopausal status, and PMH use
Samanic (2004)	4,500,700	4356 (brain)	0	0	12	1 July 1969–30 September 1996	USA	> = 18	All US veterans with at least one hospital visit and one year of follow up were at first deemed eligible. Men of a race different than black or white and women in general were excluded from the analysis due to small numbers.Also excluded were individuals whose race could not be determined (n = 62,622), individuals with discrepant records (n = 887) and individuals whose age at cohort entry was less than 18 or greater than 100 (n = 3,003)	Diagnosis of cancer according to the ICD8-A or ICD9-CM from computerized discharge records for inpatients visits at Veterans Affairs hospitals across the US. Cancers diagnosed during the first hospital visit, within the first year of the beginning of follow-up or within a year from the diagnosis of obesity were excluded from the analysis.	A discharge diagnosis of obesity (ICD8 = 277 or ICD9 = 278.0) during any visit.	Age, calendar year
Oh (2005)	781,283	234 (brain)	0	0	10	1992–31 December 2001	Korea	> = 20	Civil servants, private school workers and their dependants, who were members of the Korean National Health Insurance Corporation (KNHIC) and for whom a general health status examination is obligatory by law every 2 years. Of the possible patients men who were aged 20 years or older and had medical examination in 1992 were selected. Patients who did not respond to questionnaires or had a previous cancer were excluded.	Diagnosis of cancer including histological type were obtained from the Korea Central Cancer Registry, using each individual’s personal identification number. Where such data were not available data were obtained from the KNHIC as well. Some of the 9.1% of the cancer patients additionally identified through the KNHIC data, might have been caused either by false registration of noncancer patients or failure of real patient registration in the KCCR. Of these discrepant cancer patients, only those whose cancer was confirmed by medical bills or reports some form of treatment, or death certificates were included in the analyses. In the cases of death, multiple cancers, and others, patients were censored at the time of the first occurrence.	Data on weight and height were collected by direct measurements at medical institutions equipped with facilities and staff approved by the regulations defined by the KNHIC.	Age, smoking status, average amount of alcohol consumed per day, frequency of regular exercise for more than thirty minutes during a week, family history of cancer, residency area at baseline.
Samanic (2006)	362,552	918 (brain)	0	0	19	1971–31 December 1999	Sweden	> = 18	Workers registered in the health examination database of the Swedish Foundation for Occupational Safety and Health of the Construction Industry, between 1971 and 1992 comprised the cohort population. Women were excluded from the study due to small numbers. Also excluded were male workers with indeterminate dates of emigration or baseline examination, men with missing baseline height or weight measurements, and men younger than 18 or older than 67 years of age at baseline examination.	Linkage to the population based Swedish cancer registry via the national registration number.	Weight and height data from the baseline examination.	Attained age, attained calendar year, smoking status
Holick (2007)	237794	0	0	296	15.43	1980–2003	The Health Professionals Follow-Up Study (HPFS), the Nurses’ Health Study I (NHS I), and NHS II, USA	25–75	Nurses’ Health Study I; female nurses returned a mailed questionnaire that assessed information on lifestyle factors and medical and smoking histories. Similarly, the Health Professionals Follow-Up Study (HPFS) is a cohort of 51 529 US male physicians, dentists, optometrists, osteopaths, podiatrists, pharmacists, and veterinarians who were 40–75 y of age at enrollment in 1986. The study design and methods of dietary assessment and follow-up for the Nurses’ Health Study II (NHS II) are very similar to those of NHS I. In 1989, 116 686 women aged 25–42 y and living in 14 US states were enrolled into the NHS II. Participants who reported a history of cancer other than nonmelanoma skin cancer and those with missing information on diet at baseline were excluded.	On each biennial questionnaire, the participants were asked whether they had been diagnosed with any form of cancer. When permission was received from the case subjects (or next of kin for decedents), medical records and pathology reports were obtained from hospitals and reviewed by study investigators, who were blinded to questionnaire exposure information. Medical records were requested for reported and deceased glioma cases;88% of glioma diagnoses were confirmed by medical records. When the researchers were unable to obtain medical records, they attempted to corroborate diagnoses of glioma with additional information from the participant, next of kin, by death certificate, or by cross-linking with cancer registries. They only included case subjects for whom a medical record or other confirmation of the cancer was obtained.	Self administered questionnaire	None
Benson (2008)	1249670	1563 (CNS/brain)	390	646	6.2	May 1996–31 December 2005	UK	50–65	Million Women Study; women completed a recruitment questionnaire about reproductive factors, sociodemographic factors, and other personal characteristics.	All study participants have been flagged on the National Health Service central registers, so that tumour registrations (benign and malignant) and deaths are routinely notified to the study investigators. Incident central nervous system tumours were included from the following sites: ICD-10 C70, C71, C72.0, C75.1–3, D32, D33, D35.2–4, D42, D43, and D44.3–5. Incident cases of glioma, morphology codes ICD-O 9380–9481, and meningioma, morphology codes ICD-O 9530–9539, were identified within these sites.	Recruitment questionnaire	Height (cm), strenuous exercise, socioeconomic level, smoking, alcohol intake (g/day), parity, age at first birth (years), oral contraception use duration
Moore (2009)	499437	0	0	480	8.2	1995—December 2003	Six states (California, Florida, Pennsylvania, New Jersey, North Carolina, and Louisiana) and two metropolitan areas (Atlanta, Georgia and Detroit, Michigan), USA	50–71	NIH-AARP Diet and Health Study; AARP members completed the questionnaire satisfactorily. In late 1996, 334,908 participants responded to a second questionnaire that was mailed to those still living in a study area and having no prevalent cancer of the colon, breast, or prostate. Of the 566,402 baseline questionnaire respondents, participants whose questionnaires were completed by proxy respondents (n = 15,760) or who had a previous diagnosis of cancer (n = 51,205) were excluded. After exclusions, the analytic cohort consisted of 499,437 participants, including 305,681 persons who completed the second questionnaire.	Incident, first primary brain cancer cases (ICD-10 Edition codes C710-C719 were identified by linking the cohort with eight state cancer registries serving the cohort and three additional states (Arizona, Nevada, and Texas). Gliomas were defined as malignant brain neoplasms with microscopically confirmed ICD-O-3 histology codes between 9380 and 9480. An alternative definition was also examined; using ICD-O-3 codes 9380 to 9460, but the number of cases was the same; therefore, results were identical.	Self reported weight and height in questionnaires	Age at baseline, age-squared, gender, race, highest attained level of education, marital status, physical activity during the past 10 years, adult height, BMI at age 18 years
Parr (2010)	401,215	191 (CNS/brain)	0	0	4	1961-NR	Asia, Australia, New Zealand	> = 20	Eligible studies had a study population from the Asia-Pacific region, a prospective cohort study design, at least 5000 person-years of follow up, baseline recordings of date of birth (or age), sex and blood pressure, date of death or age at death recorded during follow up. Studies were classified as Asian if participants were recruited from mainland China, Hong Kong, Taiwan, Japan, South Korea, Singapore, or Thailand and as ANZ if participants were from Australia or New Zealand. Studies were excluded if participant entry was dependant on a particular condition or risk factor. Of the 575,458 participants of 20 years or older in the included cohorts, 26% were excluded because of missing follow up for cancer mortality, missing BMI values, or a reported BMI of less than 12 kg/m^2^ or greater than 60 kg/m^2^. Baseline data on height and weight (or BMI) and at least one cancer event were available from 39 cohorts in the APCSC database. Individuals with less than 3 years of follow up were excluded.	Fatal cases; cancer mortality was classified according to the ninth or tenth revision of the International Classification of Diseases (ICD-9 or ICD-10). ICD-7 codes reported by some of the studies were recorded into version 9 or 10 by the project secretariat. Five small studies did not use ICD codes. These studies contributed less than 5% of all cancer deaths, most of which (>80%) were grouped into a category for other or unspecified sites and included in the analysis of all cancer, or included in specified categories by the project secretariat using all available information. Summary reports were referred back to principal investigators of each collaborating study for review and confirmation. Histological subtypes were not available in the APCSC.	Baseline data on height and weight (or BMI) were available from 39 cohorts in the APCSC database.	Age, smoking status
Johnson (2011)	27791	0	125	0	10.47	1986–2004	Iowa, USA	55–69	Iowa Women’s Health Study cohort; In 1986, a questionnaire was mailed to women who were randomly selected from Iowa driver’s license files; 41,836 women returned the questionnaire (response rate, 42.7%). All women who never enrolled in both Part A (includes inpatient care in hospitals and nursing homes) and Part B (outpatient care, which has detailed claims data available starting in 1992) of Medicare for at least 1 month on or after 1 January 1993 (n = 3014) were excluded. Women who did not complete the 1992 questionnaire (n = 8819), because it contained many items used in this analysis were also excluded. Finally, women with a meningioma in Medicare claims before 1993 (n = 46) or any history of cancer or cancer chemotherapy as reported on the 1986 or 1992 questionnaires or through linkage to the Iowa SEER Cancer Registry through 1992 (n = 6723) were also excluded.	Incident meningiomas in the cohort were identified by linkage to Medicare files; meningioma cases were identified from Medicare data by using the ICD-9 Revision, codes 192.1, 192.3, 225.2, 225.4, and 237.6 (neoplasms of the cerebral or spinal meninges). To be classified as a case, the authors required at least 1 of the diagnosis codes from the MedPAR (hospital) file or any 2 diagnoses >30 days apart in the carrier or outpatient files.	Self reported via questionnaire	Age
Michaud (2011)	380775	0	203	340	8	1992–2004	Turin, Italy; Cambridge, United Kingdom; Bilthoven, Utrecht, the Netherlands; Florence, Varese, Ragusa, and Naples, Italy; Granada, Norway, Navarra, San Sebastian, Asturias and Murcia, Spain; Oxford, United Kingdom; Malmo, Umea, Sweden; Aarhus and Copenhagen, Denmark	35–70	European Prospective Investigation into Cancer and Nutrition (EPIC) cohort; France was not included in the study as there were insufficient data to distinguish histology of the brain tumor at the time of the analysis. Prevalent cancers at recruitment (except for nonmelanoma skin cancer) and individuals with no follow-up data (n = 27,082) were excluded.	Incident cases were identified through linkage to population cancer registries in Denmark, Italy, the Netherlands, Norway, Spain, Sweden, and the United Kingdom, or with a combination of methods including linkage to health insurance records, cancer and pathology registries, and active follow-up of study participants or their next of kin in France, Germany, and Greece. All primary incident cases diagnosed with glioma (coded using International Classification of Diseases- Oncology [ICD-O] second edition: 9380–9460, 9505) or meningioma (ICD-O-2 codes 9530–9537) through the end of follow-up were included. Two of the 5 centers in Spain did not record data on benign tumors and reported no meningioma cases.	Standardized questionnaires, anthropometric data measured at baseline; self reported measures corrected for bias	Age, country, sex, education
Edlinger (2012)	578,462	1236 (brain)	338	410 (high grade) 98 (low grade)	10	1972–2003 (Austria); 1972–2005 (Norway); 1972–2006 (Sweden)	Austria, Norway, Sweden	15–99	Participants in the Metabolic Syndrome and Cancer Project (Me-Can), where information was gathered from the participants of seven population-based cohorts in Austria, Norway and Sweden. Each individual’s baseline data were taken from the first (or only) health examination with complete or near complete data.	Benign as well as malignant tumors were included in the study with the corresponding ICD-7 code 193 for all tumors. Nationwide cancer registries and cause of death registries were used to identify participants who developed cancer. Benign as well as malignant tumors were included. The starting point of the follow up was 1 year after baseline.	Baseline data were collected on height, weight from the first (or only) health examination with complete or near complete data.	Sex, birth year (in decades), baseline age, smoking status
Wiedmann (2013)	74242	0	138	148	23.5	1984–2008	Nord–Trøndelag County, Norway	> = 20	Nord–Trøndelag Health Study (HUNT Study), a general health survey. Among 85 100 eligible persons, 77 310 (90.8%) returned the questionnaire that was mailed with the invitation (questionnaire 1). A total of 74 977 (88.1%) participants attended the subsequent physical examination that included standardised measurements of height and weight. At the examination, participants received a second questionnaire, including items on life-style factors and medical history, which was to be filled in at home and returned in a prestamped envelope. Information on BMI was available in 74 339 (87.4%) participants. Among these, 72 were excluded because of prevalent primary CNS tumours and 25 because of missing follow-up data. Thus, 74 242 (87.4%) individuals constituted the study population; patients with CNS tumours other than the ones that were considered as end points in the analysis were excluded at the statistical analysis.	Linkage at the Cancer Registry of Norway. Subgroups of CNS neoplasms were defined using ICD-O-3 histology codes (International Classification of Diseases for Oncology, Third Edition) 9380–9480 for gliomas, 9530–9539 for meningiomas.	Standardised measurements of height and weight were performed at the baseline clinical examination and BMI was calculated	Age and in combined models sex

NR: not reported, NA: Not Applicable

**Table 2:** Characteristics of the included case-control studies.

Study (year)	Brain/CNS cancer cases	Meningioma cases	Glioma cases	Number of controls	Study period	Region	Age range	Definition/features of Cases	Definition of Controls	Matching factors	Type of ascertainment of BMI	Adjusting factors
Pan (2004)	1009	0	0	5039	1994–1997	The Canadian provinces of Alberta, British Columbia, Manitoba, Newfoundland, Nova Scotia, Ontario, Prince Edward Island and Saskatchewan.	20–76	Subjects participating in the National Enhanced Cancer Surveillance System (NECSS) with a newly diagnosed, histologically confirmed primary cancer, who after the consent of their physician were sent a questionnaire via mail or a later telephone interview if so needed.	The NECSS used frequency matching to the overall case group with similar age and sex distributions in the selection of population controls, so that there would be at least one control for every case within each sex and 5-year age group for any specific cancer site within each province. The sampling strategy for control selection varied by province depending on data availability, data quality (completeness and timeliness), and the confidentiality restrictions of provincial databases.	Age, gender	Data on weight and height from questionairre	5-year age group, province of residence, education, pack-years of smoking, alcohol drinking, total caloric intake, vegetable intake, dietary fiber intake, and recreational physical activity, for women adjustment was made also for: menopausal status, number of livebirths, age at menarche, and age at end of first pregnancy
Custer (2006)	0	143	0	286	January 1, 1995 and June 30, 1998	King, Pierce and Snohomish counties of western Washington State, USA	> = 18	Cases with incident intracranial meningioma, histologically confirmed were identified using the National Cancer Institute's Surveillance, Epidemiology, and End Results program for King, Pierce and Snohomish counties of western Washington State.	Using random-digit dialing or Medicare eligibility lists, two controls for each case were recruited.	Gender, age within 5 years, and county of residence	Structured in-person interview and questionnaire	Age, education
Lee (2006)	0	219	0	260	1987–1993	Chicago, IL, USA	NR	Females diagnosed with histologically confirmed incident meningioma were identified from three medical institutions, using medical records: Chicago Institute of Neurosurgery and Neuroscience (CINN), University of Illinois Medical Center (UIC) and Loyola University Medical Center (LUMC). Questionnaires were sent to all study subjects. Of 341 cases to whom letters were mailed, 87 could not be included, leaving a total of 254 available to complete the questionnaire. Reasons for these 87 exclusions were as follows: 22 were dead, 9 were too ill to respond, 11 did not know English, 1 did not live in the United States and 41 had undeliverable addresses. Since the focus was on hormonal factors, another 3 cases were excluded because the meningioma was diagnosed within a year of the initiation of menses. Cases were additionally excluded as they refused to participate, did not respond, or had a poor quality of response.	Female spouses of male back pain patients were targeted as controls. All male back pain patients from clinics in these same institutions whose medical records indicted that they were married at the time of their treatment were selected from the same year as the cases. A total of 647 male, married, back pain patients were contacted. Once contacted, 302 were not eligible for the following reasons: 124 back pain patients did not have female partners, 46 were dead, 15 were too ill to respond, 38 did not know English, 6 did not live in the United States, and 73 had undeliverable addresses. Of the 345 remaining potential controls, 260 returned completed questionnaires after 3 mailings and a telephone follow-up. Controls were additionally excluded as they refused to participate, did not respond, or had a poor quality of response.	Referral population	Self administrated questionnaires; telephone interviews based on the same questionnaires; the questionnaire was pilot tested in a clinic of one of the collaborating institutions (CINN)	None
Aghi (2007)	0	32	32	32	2001–2005	Massachusetts, USA	NR	Male patients who underwent initial craniotomy for benign meningiomas or high-grade gliomas at Massachusetts General Hospital; patients were identified and records obtained from searching both a departmental computerized database and hospital computerized records. Five patients with multiple meningiomas were excluded from the analysis. Meningioma and high-grade glioma diagnoses were confirmed on histological analysis of permanent sections of surgical specimens.	From the same two databases, control subjects, men undergoing initial craniotomy for unruptured aneurysms during the same time period, were identified.	Year of surgery (gliomas); none (meningiomas)	BMI was calculated using the information obtained during routine preoperative patient testing with the same calibrated weighing scale and ruler for each patient	None
Cabaniols (2011)	0	0	122	59	January-December 2005	Marseille and the Sainte Anne’s hospitals of Toulon, France	> = 18	Patients of at least 18 years of age, with residence within the administrative region of Provence-Alpes-Cote d’ Azur (PACA) with a diagnosis of previously untreated glioma grades II to IV according to the WHO classification criteria, who had provided a fully informed consent and have agreed to fill in the given questionnaire. Pilocytic astrocytomas (WHO grade I) were excluded from the study since they mainly concern pediatric population, also excluded were cases of recurrence of previous malignant primitive brain tumors.	Controls were residents of the administrative region of PACA, hospitalized for reasons unrelated to cancer (namely herniated intervertebral disk, intracranial aneurysm, neurosurgical traumatism requiring surgery and epidural hematoma) and selected randomely from the neurosurgical department of the hospital.	Age, sex	Standardized, structured questionnaire data on size and weight were used to calculate each patients BMI.	Age, sex
Claus (2013)	0	1127	0	1092	May 1 2006—October 6 2011	Connecticut, Massachusetts, and North Carolina as well as the California counties of Alameda, San Francisco, Contra Costa, Marin, San Mateo, and Santa Clara and the Texas counties of Brazoria, Fort Bend, Harris, Montgomery, Chambers, Galveston, Liberty, and Waller counties of Texas, USA	20–79	Histologically confirmed intracranial meningioma; female patients were identified through the Rapid Case Ascertainment systems and state cancer registries of the respective sites; Six hundred ninety-six patients were ineligible due to out-of-state residency (n = 48), language (n = 74), recurrent meningioma (n = 84), incarceration (n = 3), age (n = 50), spinal meningioma (n = 148), pathology unavaiable for review (n = 75), mental or medical (for example, deafness) illness (n = 110), death (cause of death other than meningioma) (n = 79), another pathology (for example, lung metastasis) (n = 16), or other reason (n = 9).	Control individuals were selected by random-digit dialing by an outside consulting firm (Kreider Research). Individuals were English- or Spanish-language speaking. One hundred ten control individuals were ineligible due to out-of-state residency (n = 6), language (n = 8), a history of brain tumor of unknown pathology (n = 8), age group (n = 1), mental or medical illness (n = 70), death (n = 3), or other reason (n = 14).	5-year age interval, sex, and state of residence	Consent forms and questionnaire	age, race, education, menopause status, age at menopause, age at menarche, number of full-term pregnancies, age at first live birth, ever use of oral contraceptives, ever use of hormone replacement therapy, ever use of fertility meds, smoking, alcohol use, breastfeeding
Little (2013)	0	0	1111	1096	December 2004—June 2012	Southeastern United States [Southeastern United States including Vanderbilt University Medical Center (Nashville, TN), Moffitt Cancer Center (Tampa, FL), University of Alabama at Birmingham (Birmingham, AL), Emory University (Atlanta, GA), and the Kentuckiana Cancer Institute (Louisville, KY)]	25–92	Recent (within 3 months) primary diagnosis of glioma. Glioma cases were identified in neurosurgery and neuro-oncology clinics in the Southeastern United States.	Controls were identified from white page listings. An estimated 50% of contacted eligible households yielded a participating control.	Age, gender, race, and state of residence	Self-reported weight and height, reliability check of responses	Age, race, education, state of residence, and in combined models, gender
Schildkraut (2014)	0	456	0	452	May 1 2006 –October 9 2012	Connecticut, Massachusets, and North Carolina, as well as the Alameda San Fransisco, Contra Costa, Marin, San Mateo, and Santa Clara counties of California, and the Brazoria, Fort Bend, Harris, Montgomery, Chambers, Galveston, Liberty and Waller counties of Texas, USA	20–79	Male patients with a histologically confirmed intracranial meningioma, speaking English or Spanish. Study patients with previous meningioma and/or a brain lesion of unknown type were excluded. Identification was made through the Rapid Case Ascertainment Systems and the respective states’ cancer registries. 63% of the eligible cases participated in the interview. A total of 756 cases were ineligible due to out of state residency (n = 47), language (n = 82), recurrent meningioma (n = 85), incarceration (n = 3), age (n = 50), spinal meningioma (n = 154), pathological specimen unavailable for review (n = 80), mental or medical illness (n = 130), death (n = 97), another pathology (such as lung metastasis, n = 18) or other (n = 10). An additional 1127 cases were excluded because they were females.	Control individuals were selected by an outside consulting firm (Kreider) using random digit dialing. 50% of the eligible controls participated in the interview. Among them 124 were ineligible due to out of state residency (n = 6), language (n = 8), a history of previous brain tumor of unknown pathology (n = 9), age group (n = 3) mental or medical illness (n = 79), death (n = 4) or other (n = 15) An additional 1902 controls were excluded because they were females.	Age, race	Telephone interview	Age, race

NR: not reported

### Meta-analysis: brain/CNS tumors

**Fig 2:**
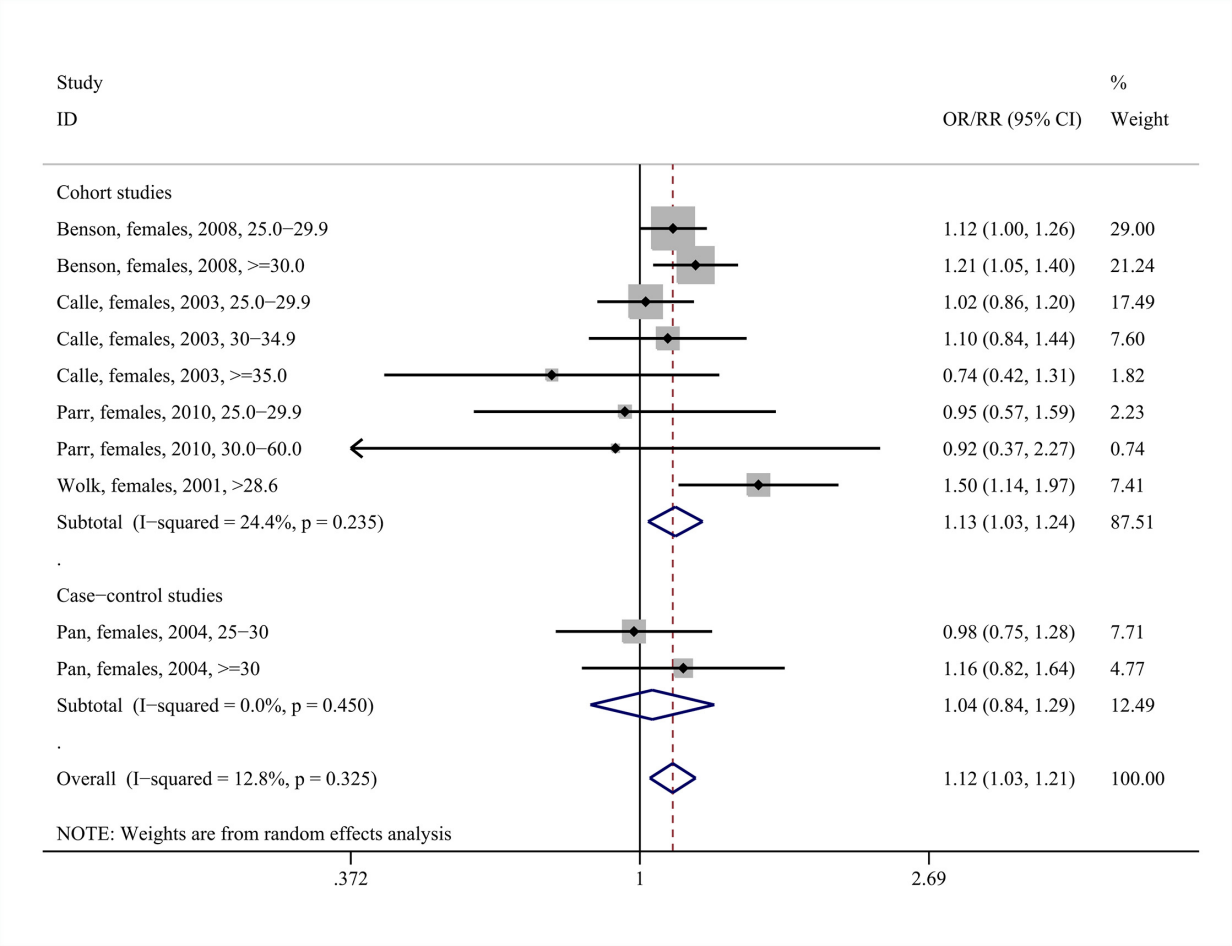
Forest plot describing the association between overweight status/obesity and brain/CNS tumor risk among females. Apart from the overall analysis, the subanalyses on cohort (upper panels) and case-control (lower panels) studies are presented.

The middle panels of Table 3 present the results of the meta-analyses regarding the association between being overweight/obese and risk of overall brain/CNS tumors. Overweight status/obesity was associated with increased risk for brain/CNS tumors among females (pooled RR = 1.12, 95%CI: 1.03–1.21, 10 study arms, I^2^ = 12.8%, Fig 2), with the association being evident in the subset of cohort studies (pooled RR = 1.13, 95%CI: 1.03–1.24, eight study arms, I^2^ = 24.4%). The positive association was mainly due to the obese female subjects, as increased brain/CNS tumor risk was observed among them (pooled RR = 1.19, 95%CI: 1.05–1.36, six study arms, I^2^ = 17.8%, Fig A in S1 File).

**Table 3:** Results of the meta-analyses examining the association between obesity and risk brain/CNS tumors, meningiomas and gliomas. Bold cells denote statistically significant associations.

		Overweight and obese pooled together			Overweight			Obese		
		n^§^	Effect estimate (95%CI)	Heterogeneity I^2^, p, τ^2^	n^§^	Effect estimate (95%CI)	Heterogeneity I^2^, p, τ^2^	n^§^	Effect estimate (95%CI)	Heterogeneity I^2^, p, τ^2^
**Brain/CNS tumors**	Females	10	**1.12 (1.03–1.21)**	12.8%, 0.325, 0.002	4	1.07 (0.98–1.17)	0.0%, 0.672, <0.0001	6	**1.19 (1.05–1.36)**	17.8%, 0.298, 0.005
	*Cohort studies*	8	**1.13 (1.03–1.24)**	24.4%, 0.235, 0.0041	3	1.08 (0.99–1.19)	0.0%, 0.583, <0.0001	5	**1.19 (1.01–1.40)**	33.7%, 0.196, 0.0116
	*Case-control studies*	2	1.04 (0.84–1.29)	0.0%, 0.450, <0.0001	1	Only one study	NC	1	Only one study	NC
									
	Males	14	1.01 (0.94–1.08)	12.1%, 0.321, 0.0022	6	1.01 (0.93–1.10)	0.0%, 0.596, <0.0001	8	1.01 (0.87–1.17)	36.1%, 0.141, 0.0144
	*Cohort studies*	12	0.99 (0.91–1.08)	20.3%, 0.244, 0.0043	5	1.00 (0.92–1.10)	0.0%, 0.485, <0.0001	7	1.00 (0.84–1.18)	40.9%, 0.119, 0.0189
	*Case-control studies*	2	1.08 (0.91–1.27)	0.0%, 0.760, <0.0001	1	Only one study	NC	1	Only one study	NC
									
	Study arms not distinguishing sexes	7	1.09 (0.97–1.23)	43.9%, 0.098, 0.0102	3	1.01 (0.90–1.13)	0.0%, 0.758, <0.0001	4	1.19 (0.99–1.44)	53.1%, 0.094, 0.0186
	*Cohort studies*	5	1.09 (0.90–1.31)	59.9%, 0.041, 0.0247	2	0.96 (0.82–1.14)	0.0%, 0.910, <0.0001	3	1.19 (0.88–1.59)	68.7%, 0.041, 0.0432
	*Case-control studies*	2	1.09 (0.96–1.24)	0.0%, 0.394, <0.0001	1	Only one study	NC	1	Only one study	NC
**Meningiomas**	Females	16	**1.27 (1.13–1.43)**	35.7%, 0.078, 0.0188	9	1.11 (0.99–1.25)	8.3%, 0.366, 0.0029	7	**1.48 (1.28–1.71)**	0.0%, 0.546, <0.0001
	*Cohort studies*	10	**1.39 (1.17–1.64)**	39.9%, 0.092, 0.0277	5	1.19 (0.97–1.45)	27.1%, 0.241, 0.0145	5	**1.60 (1.33–1.93)**	0.0%, 0.539, <0.0001
	*Case-control studies*	6	**1.15 (1.00–1.32)**	10.4%, 0.349, 0.0032	4	1.06 (0.91–1.24)	0.0%, 0.446, <0.0001	2	**1.33 (1.07–1.66)**	0.0%, 0.590, <0.0001
									
	Males	9	**1.58 (1.22–2.04)**	27.2%, 0.202, 0.038	4	1.39 (0.95–2.03)	29.0%, 0.238, 0.0457	4	**1.78 (1.22–2.61)**	29.6%, 0.224, 0.0537
	*Cohort studies*	4	1.11 (0.74–1.64)	0.0%, 0.757, <0.0001	2	1.03 (0.64–1.66)	0.0%, 0.603, <0.0001	2	1.30 (0.64–2.62)	0.0%, 0.427, <0.0001
	*Case-control studies*	5	**1.86 (1.40–2.48)**	24.2%, 0.260, 0.0256	2	**1.72 (1.22–2.42)**	0.0%, 0.320, <0.0001	2	**2.04 (1.22–3.42)**	51.4%, 0.128, 0.0995
									
	Study arms not distinguishing sexes	6	**1.31 (1.12–1.53)**	0.0%, 0.846, <0.0001	3	1.22 (0.98–1.50)	0.0%, 0.645, <0.0001	3	**1.44 (1.13–1.84)**	0.0%, 0.969, <0.0001
	*Cohort studies*	6	**1.31 (1.12–1.53)**	0.0%, 0.846, <0.0001	3	1.22 (0.98–1.50)	0.0%, 0.645, <0.0001	3	**1.44 (1.13–1.84)**	0.0%, 0.969, <0.0001
	*Case-control studies*	0	-	-	0	-	-	0	-	-
**Gliomas**	Females	6	**1.17 (1.03–1.32)**	0.0%, 0.757, <0.0001	3	**1.19 (1.02–1.38)**	0.0%, 0.611, <0.0001	3	1.13 (0.92–1.38)	0.0%, 0.472, <0.0001
	*Cohort studies*	4	**1.14 (1.00–1.29)**	0.0%, 0.824, <0.0001	2	1.17 (0.99–1.37)	0.0%, 0.463, <0.0001	2	1.09 (0.88–1.34)	0.0%, 0.757, <0.0001
	*Case-control studies*	2	**1.48 (1.00–2.20)**	0.0%, 0.675, <0.0001	1	Only one study	NC	1	Only one study	NC
									
	Males	6	0.96 (0.76–1.23)	22.4%, 0.265, 0.0199	3	1.03 (0.84–1.28)	0.0%, 0.383, <0.0001	3	0.81 (0.42–1.57)	49.5%, 0.138, 0.942
	*Cohort studies*	2	0.93 (0.67–1.28)	0.0%, 0.364, <0.0001	1	Only one study	NC	1	Only one study	NC
	*Case-control studies*	4	0.90 (0.57–1.40)	43.8%, 0.149, 0.0836	2	1.14 (0.88–1.48)	0.0%, 0.568, <0.0001	2	0.44 (0.09–2.28)	57.0%, 0.127, 0.157
									
	Study arms not distinguishing sexes	14	1.03 (0.94–1.14)	0.0%, 0.556, <0.0001	7	1.02 (0.89–1.18)	17.6%, 0.296, 0.0062	7	1.03 (0.87–1.22)	0.0%, 0.626, <0.0001
	*Cohort studies*	11	1.01 (0.90–1.13)	0.0%, 0.697, <0.0001	5	0.99 (0.86–1.15)	0.0%, 0.581, <0.0001	6	1.03 (0.86–1.23)	0.0%, 0.499, <0.0001
	*Case-control studies*	3	1.04 (0.78–1.40)	45.7%, 0.158, 0.0317	2	0.98 (0.57–1.68)	72.5%, 0.057, 0.1152	1	Only one study	NC

^§^number of study arms; NC: not calculable

On the other hand, no association between overweight status/obesity and brain/CNS tumor risk was identified in males (pooled RR = 1.01, 95%CI: 0.94–1.08, 14 study arms, I^2^ = 12.1%, Fig B in S1 File). Subgroup analyses by overweight or obesity status replicated the null association (Fig C in S1 File).

In the instances where studies did not distinguish their findings by gender (referred to as “study arms which did not distinguish sexes”), the pooled analysis did not yield any statistically significant association regarding brain/CNS tumor risk (pooled RR = 1.09, 95%CI: 0.97–1.23, seven study arms, I^2^ = 43.9%, Figs D and E in S1 File). Most probably, this was due to the admixture of the two genders, with their differing profiles.

A *post hoc* sensitivity analysis including studies reporting exclusively on brain tumor risk (therefore excluding the study arms with collective reporting on “brain/CNS” as a whole) was hampered by the smaller number of eligible study arms; no significant associations were noted therein (Fig F in S1 File).

### Meta-analysis: meningiomas

**Fig 3:**
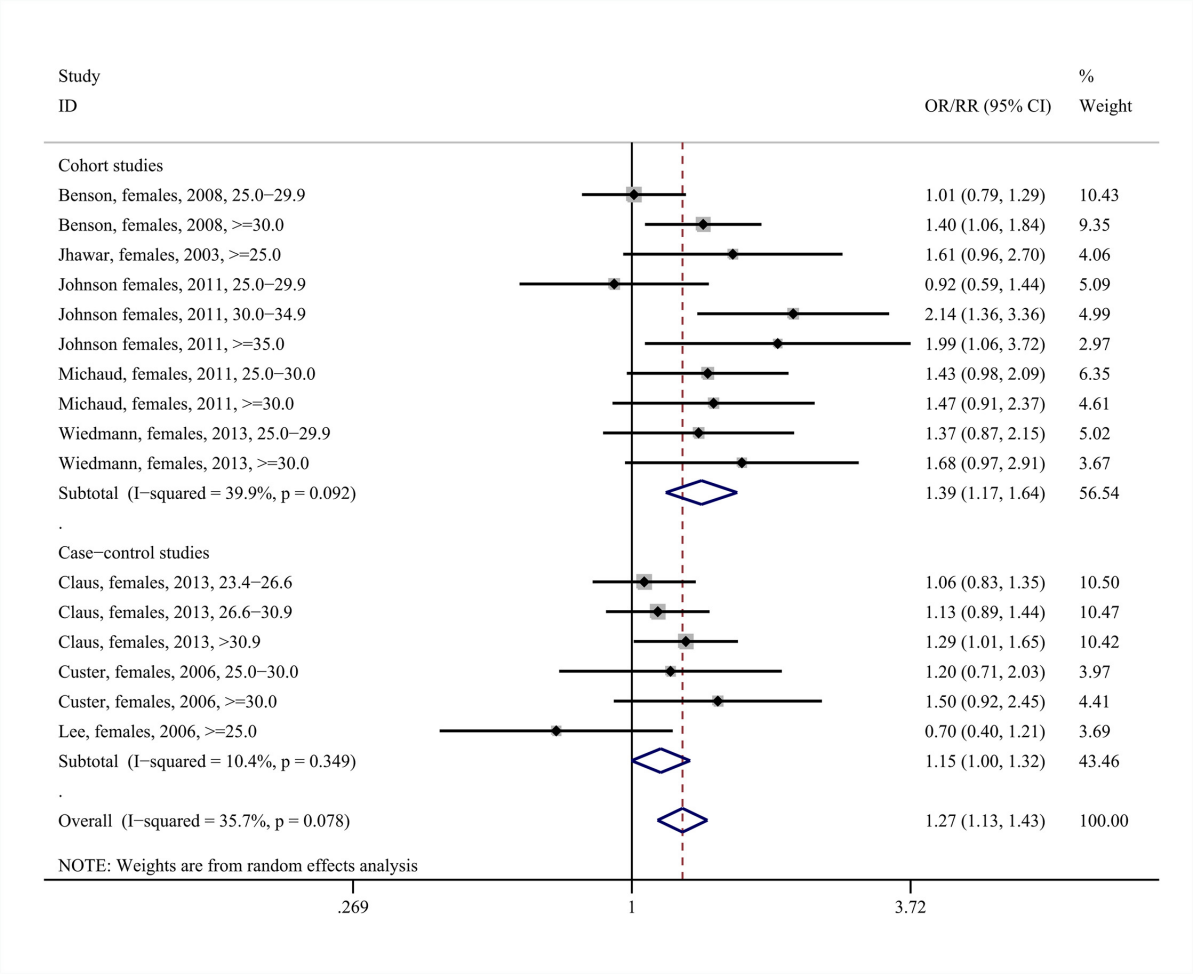
Forest plot describing the association between overweight status /obesity and meningioma risk among females,. Apart from the overall analysis, the subanalyses on cohort (upper panels) and case-control (lower panels) studies are presented.

The middle panels of Table 3 present the results of the meta-analyses regarding the association between overweight status/obesity and risk of meningioma. Overweight status/obesity was associated with increased risk for meningioma among females (pooled RR = 1.27, 95%CI: 1.13–1.43, 16 study arms, I^2^ = 35.7%, Fig 3), with the association replicated both in the subset of cohort (pooled RR = 1.39, 95%CI: 1.17–1.64, 10 study arms, I^2^ = 39.9%) as well as case-control studies. Once again, the positive association could be mainly attributed to the obese female subjects, as increased meningioma risk was noted among them (pooled RR = 1.48, 95%CI: 1.28–1.71, seven study arms, I^2^ = 0.0%, Fig G in S1 File); once again the correlation was reproducible upon the subgroups of cohort as well as case-control studies. On the other hand, the overweight females presented only with a marginal trend towards increased risk for meningioma (pooled RR = 1.11, 95%CI: 0.99–1.25, p = 0.085, nine study arms, I^2^ = 8.3%, Fig G in S1 File).

**Fig 4:**
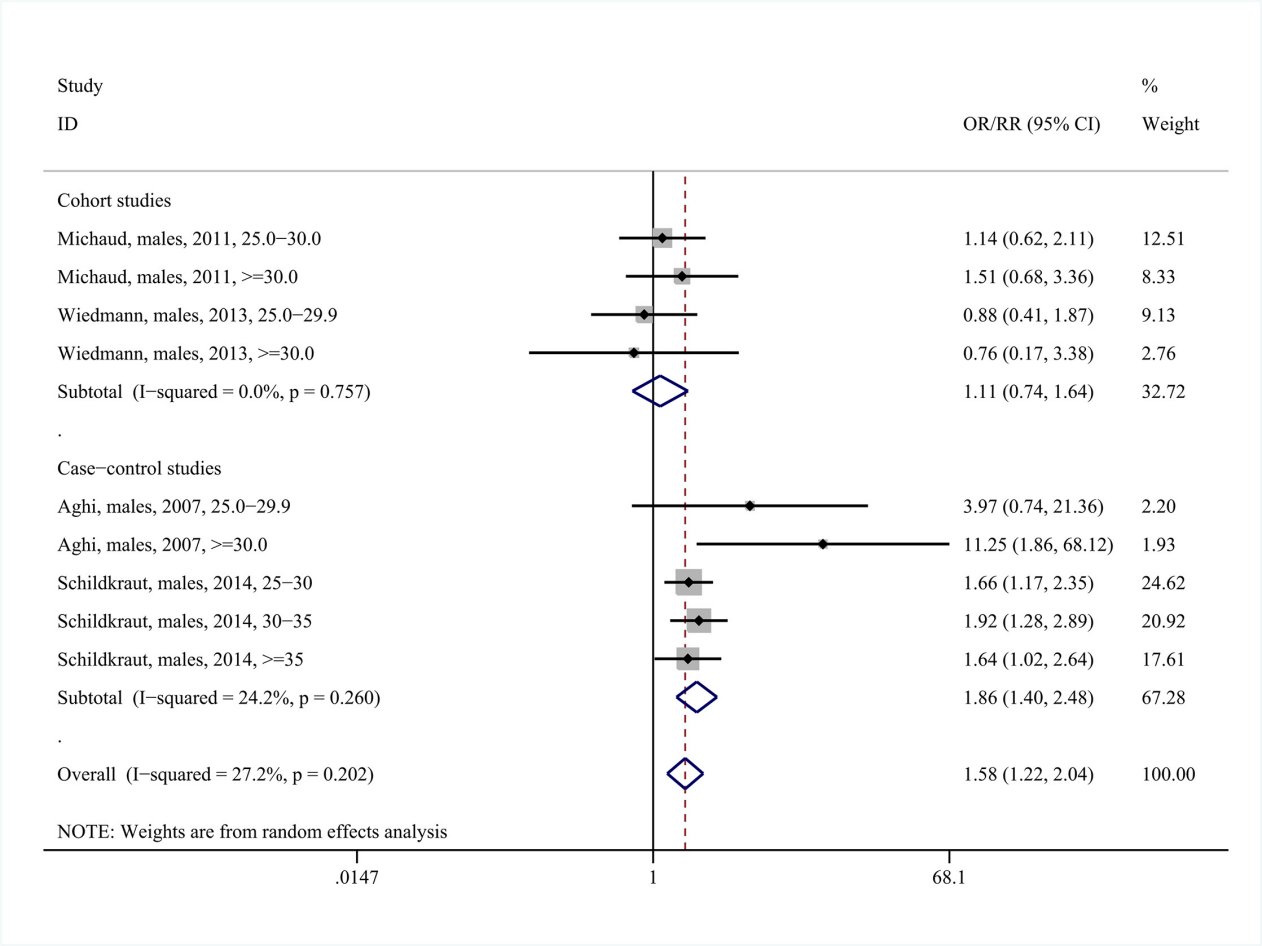
Forest plot describing the association between overweight status /obesity and meningioma risk among males. Apart from the overall analysis, the subanalyses on cohort (upper panels) and case-control (lower panels) studies are presented.

The results among males were compatible with those among females. Overweight status/obesity was associated with increased risk for meningioma among males (pooled RR = 1.58, 95%CI: 1.22–2.04, nine study arms, I^2^ = 27.2%, Fig 4); however, the positive association appeared to be confined to the case-control studies. The positive association was mainly attributable to the subgroup of obese males (pooled RR = 1.78, 95%CI: 1.22–2.61, four study arms, I^2^ = 29.6%, Fig H in S1 File).

As expected, the positive association between overweight status/obesity and meningioma risk was also noted during the pooling of the six study arms that did not distinguish between the two sexes (pooled RR = 1.31, 95%CI: 1.12–1.53, I^2^ = 0.0%, Fig I in S1 File).

### Meta-analysis: gliomas

**Fig 5:**
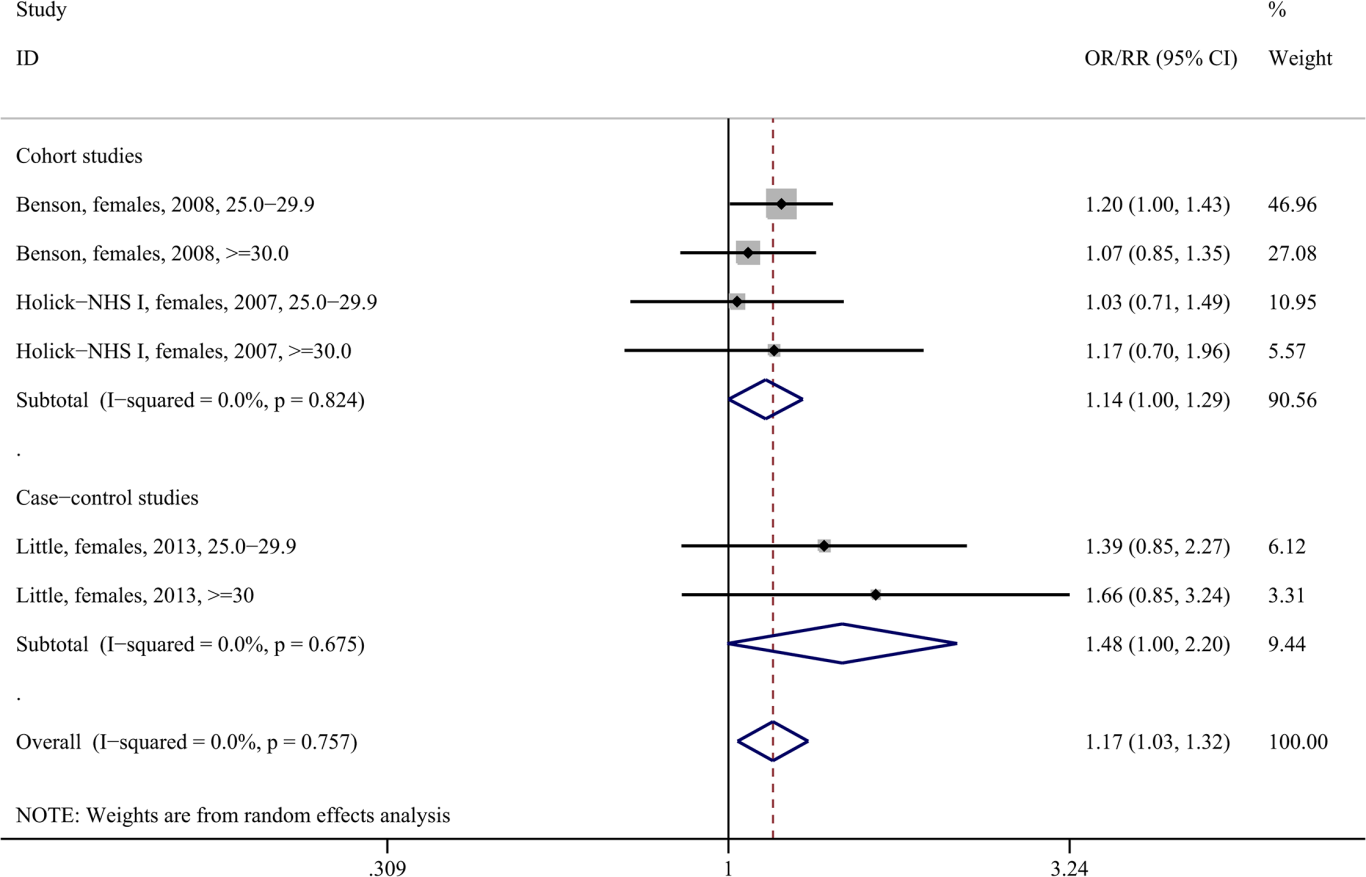
Forest plot describing the association between overweight status/obesity and glioma risk among females. Apart from the overall analysis, the subanalyses on cohort (upper panels) and case-control (lower panels) studies are presented.

The lower panels of Table 3 present the results of the meta-analyses regarding the association between overweight status/obesity and glioma risk. Being overweight or obese correlated with increased risk for glioma among females (pooled RR = 1.17, 95%CI: 1.03–1.32, six study arms, I^2^ = 0.0%, Fig 5), with the association spanning both the subsets of cohort (pooled RR = 1.14, 95%CI: 1.00–1.29, four study arms, I^2^ = 0.0%) as well as case-control studies. Statistical significance was achieved regarding overweight females (pooled RR = 1.19, 95%CI: 1.02–1.38, three study arms, I^2^ = 0.0%, Fig J-i in S1 File), whereas the association among obese females did not reach significance (pooled RR = 1.13, 95%CI: 0.92–1.38, I^2^ = 0.0%, Fig J-ii in S1 File); of note though only three study arms reported on the latter subgroup, denoting the scarcity of data.

Among males, there was no association between being overweight/obese and glioma risk (pooled RR = 0.96, 95%CI: 0.76–1.23, six study arms, I^2^ = 22.4%, Fig K in S1 File); the null pattern was reproduced at the subgroup analyses on cohort studies, case-control studies, overweight and obese subjects (Fig L in S1 File). The pooling of the 14 study arms that did not distinguish between the two sexes pointed to a null association (pooled RR = 1.03, 95%CI: 0.94–1.14, I^2^ = 0.0%, Fig M in S1 File); the null pattern was replicated at the subgroup analyses on cohort studies, case-control studies, overweight and obese subjects (Fig N in S1 File).

### Dose-response meta-regression analysis

The results of the dose-response meta-regression analysis are presented in Table A in S1 File. The positive association between overweight status/obesity and risk for meningioma among females was reflected upon the findings of the meta-regression analysis (exponentiated coefficient = 1.15, 95%CI: 1.03–1.28, BMI in increments of 5 kg/m^2^, p = 0.018 following the approach by Berlin et al. [26], Fig O in S1 File), with similar results according to the approach by Il’yasova et al. [27].

On the other hand, the remaining sets of meta-regression analyses did not yield any significant associations; of note, however, it should be noted that the majority of these meta-regression analyses should be deemed explorative, as 10 study arms represent a minimum requirement for satisfactory power according to the Cochrane Handbook [23].

### Evaluation of quality of studies and risk of bias

The evaluation of quality of studies is presented in Table B in S1 File for cohort studies and in Table C in S1 File for case-control studies. The quality of cohort studies was mainly compromised by the fact that the ascertainment of BMI was based on questionnaires rather than actual measurements. Regarding the case-control studies, their quality was often compromised by the lack of comparability of non-response rates among cases and controls, as well as the definition of controls. Regarding the overall brain/CNS analysis (Fig P in S1 File), publication bias was not significant at the overall (overweight and obese, pooled together) analysis on females (p = 0.429), males (p = 0.238), or study arms not distinguishing between the two sexes (p = 0.971). Similarly, lack of publication bias was noted regarding meningioma (p = 0.119; p = 0.761; p = 0.696, respectively, Fig Q in S1 File), whereas concerning glioma publication bias was noted only in the analysis pertaining to study arms not distinguishing between the two sexes (p = 0.421; p = 0.120; p = 0.030, respectively, Fig R in S1 File).

## Discussion

This meta-analysis highlights obesity as a risk factor for overall brain/CNS, meningiomas and gliomas in females. More specifically, being overweight or obese conferred a substantial increase in risk for all three conditions (12%, 27% and 17%, respectively). Moreover, overweight status or obesity also emerged as a risk factor for meningioma in males, conferring an excess risk of 58%.

From a methodological point of view, dose-response meta-regression analysis was often hampered by the small number of eligible study arms; nevertheless, it was capable of further validating the association between being overweight/obese and increased risk of meningioma among females. Indeed, the dose-response pattern may reinforce the causal nature of the association, according to the Bradford-Hill criteria [51]. Apart from the meta-regression analysis, the dose-response pattern could also be indirectly observed, given that obese males and females seemed sizably more burdened than overweight ones in terms of meningioma risk. Further commenting on methodological issues, this meta-analysis clearly underlines the need for separate reporting by gender in the forthcoming studies, as the study arms that did not distinguish between the two sexes seemed to merely reflect the effects of subjects’ admixture.

Obesity may act through a variety of cancer-promoting pathways, such as chronic insulin resistance, hyperinsulinemia and increased activity of insulin-like growth factor (IGF)-I [52]. Importantly, more than half of meningiomas express the IGF-1 receptor, with IGF-I inducing the growth of meningioma cells in culture; this may provide a direct mechanism of potential tumor-promoting action in both genders [53]. Similarly, IGF-I receptors have been identified in glioma cells, possessing mitogenic [54] and anti-apoptotic [55] actions; this physiological background, shared by meningiomas and gliomas, may provide evidence for the biological plausibility of the associations observed by this meta-analysis.

The underlying mechanisms regarding the sex-specific pattern regarding gliomas and overall brain/CNS tumors remain unclear, but may encompass sex hormone-related effects. The well established, positive correlation between body fat mass and the levels of estrogen [56], highlighting the role of adipose tissue as a highly active endocrine organ, seems of special importance in this context. Being overweight/obese was consistently associated with meningioma risk in females, among both subgroups (cohort and case-control studies), whereas the association pertaining to males seemed mainly due to case-control studies. Sex steroid hormone receptors are indeed present in the majority of meningiomas, namely 80 to 90% of meningiomas express the progesterone receptor and 40% express the estrogen receptor [57]; this fact is consistent with the higher incidence of meningiomas among women compared to men [4, 58], as well as the progression of meningiomas during pregnancy [59].

Limitations of our meta-analysis essentially reflect the shortcomings of individual studies. Unadjusted effect estimates have been occasionally calculated, suffering essentially from confounding, whereas gender-specific effect estimates were not always provided, as discussed earlier. Furthermore, the number of eligible study arms regarding males was substantially smaller than that regarding females, potentially limiting the power of the analyses. Moreover, heterogeneity is very often present but may remain undetected, especially for small meta-analyses [60], as was occasionally the case in the present effort. Regarding the external generalizability of the findings documented herein, it seems worth mentioning that only two studies on Chinese/Asian populations were eligible [37, 38] and they reported on brain/CNS tumors, a fact which underlines the need for relevant studies especially regarding histotypes (meningioma/glioma), so as to shed light into race-specific effects, if any. In cohort studies, the ascertainment of BMI was based on questionnaires rather than actual measurements, a fact which may have introduced reporting bias; case-control studies often suffered by the differential non-response rates among cases and controls and are inherently prone to reverse causation.

It would also be tempting to envisage future studies assessing more elaborate indices of obesity, such as the waist to hip ratio as a marker of central, obesity, or measurements of subcutaneous fat, and whole body fat proportion in an attempt to provide further insight for the underlying pathophysiological links. Future studies may also broaden the perspective of this meta-analysis, examining the associations of obesity with schwannomas and other CNS tumor types, regarding which there is currently limited data. Individual patient data (IPD) pooled analyses seem also particularly desirable, as they might effectively investigate mediating and moderating effects regarding both patient and study characteristics, whereas they might effectively account for missing data as well as minimize or more adequately explain heterogeneity.

Despite the limitations, this meta-analysis bears certain strengths that can also be acknowledged. The lack of publication bias might be one of them; nevertheless, tests for publication bias are rather underpowered, especially in the context of less than 10 studies [61]. Other assets include the broad search algorithm, the meticulous “snowball” procedure for the maximization of eligible articles and synthesized information, as well as the rigorous contact [62] with the authors who subsequently provided us with supplementary data. Indeed, for instance regarding meningioma, our meta-analysis collectively synthesized 16 study arms, whereas the most recent meta-analysis on the field focused only on six studies [22], underlying the comprehensiveness of our approach, which may well have supplied us with substantial statistical power to detect the associations, contrary to the previous work. Another strength of this meta-analysis pertains to the fact that the synthesized studies are relatively recent, published between 2001 and 2014. From a public health perspective, the association between obesity and risk of the CNS tumors seems extremely meaningful, as the modifiable nature of obesity [63] may provide opportunities for prevention regarding these rare, yet considered unpreventable, tumor types.

In conclusion, this meta-analysis is the first to highlight obesity as a risk factor for overall brain/CNS tumors, meningiomas and gliomas among females, as well as for meningiomas among males. Further accumulation of data seems mandatory regarding more elaborate obesity indices. These results open novel perspectives in the prevention of CNS tumors and warrant further investigation regarding the mechanisms underlying the pathophysiological links and potential sex-specific effects.

## Supporting Information

S1 Checklist PRISMA Checklist. (DOC)

S1 Dataset Dataset underlying the study findings. (XLSX)

S1 File Supporting Information Methods, Results, Tables and Figures. (DOCX)

## References

[1] Ohgaki H and Kleihues P (2005) Epidemiology and etiology of gliomas. Acta Neuropathol 109: 93–108. 15685439 10.1007/s00401-005-0991-y

[2] Longstreth WT Jr., Dennis LK, McGuire VM, Drangsholt MT and Koepsell TD (1993) Epidemiology of intracranial meningioma. Cancer 72: 639–648. 8334619 10.1002/1097-0142(19930801)72:3<639::aid-cncr2820720304>3.0.co;2-p

[3] Sanson M and Cornu P (2000) Biology of meningiomas. Acta Neurochir (Wien) 142: 493–505. 10898356 10.1007/s007010050462

[4] Adami HO, Hunter D and Trichopoulos D (2008) Textbook of Cancer Epidemiology. NY: Oxford University Press.

[5] Umansky F, Shoshan Y, Rosenthal G, Fraifeld S and Spektor S (2008) Radiation-induced meningioma. Neurosurg Focus 24: E7. 10.3171/FOC/2008/24/5/E7 18447746

[6] Navas-Acien A, Pollan M, Gustavsson P and Plato N (2002) Occupation, exposure to chemicals and risk of gliomas and meningiomas in Sweden. Am J Ind Med 42: 214–227. 12210690 10.1002/ajim.10107

[7] Benson VS, Pirie K, Green J, Casabonne D and Beral V (2008) Lifestyle factors and primary glioma and meningioma tumours in the Million Women Study cohort. Br J Cancer 99: 185–190. 10.1038/sj.bjc.6604445 18560401 PMC2453038

[8] Little MP, Rajaraman P, Curtis RE, Devesa SS, Inskip PD, Check DP, et al (2012) Mobile phone use and glioma risk: comparison of epidemiological study results with incidence trends in the United States. BMJ 344: e1147 10.1136/bmj.e1147 22403263 PMC3297541

[9] Linos E, Raine T, Alonso A and Michaud D (2007) Atopy and risk of brain tumors: a meta-analysis. J Natl Cancer Inst 99: 1544–1550. 17925535 10.1093/jnci/djm170

[10] Mandelzweig L, Novikov I and Sadetzki S (2009) Smoking and risk of glioma: a meta-analysis. Cancer Causes Control 20: 1927–1938. 10.1007/s10552-009-9386-z 19568697

[11] Inskip PD, Mellemkjaer L, Gridley G and Olsen JH (1998) Incidence of intracranial tumors following hospitalization for head injuries (Denmark). Cancer Causes Control 9: 109–116. 9486470 10.1023/a:1008861722901

[12] Cowppli-Bony A, Bouvier G, Rue M, Loiseau H, Vital A, Lebailly P, et al (2011) Brain tumors and hormonal factors: review of the epidemiological literature. Cancer Causes Control 22: 697–714. 10.1007/s10552-011-9742-7 21359526

[13] Claus EB, Calvocoressi L, Bondy ML, Schildkraut JM, Wiemels JL and Wrensch M (2011) Family and personal medical history and risk of meningioma. J Neurosurg 115: 1072–1077. 10.3171/2011.6.JNS11129 21780859 PMC3241000

[14] Custer BS, Koepsell TD and Mueller BA (2002) The association between breast carcinoma and meningioma in women. Cancer 94: 1626–1635. 11920521 10.1002/cncr.10410

[15] Renehan AG, Tyson M, Egger M, Heller RF and Zwahlen M (2008) Body-mass index and incidence of cancer: a systematic review and meta-analysis of prospective observational studies. Lancet 371: 569–578. 10.1016/S0140-6736(08)60269-X 18280327

[16] Wolin KY, Carson K and Colditz GA (2010) Obesity and cancer. Oncologist 15: 556–565. 10.1634/theoncologist.2009-0285 20507889 PMC3227989

[17] Ning Y, Wang L and Giovannucci EL (2010) A quantitative analysis of body mass index and colorectal cancer: findings from 56 observational studies. Obes Rev 11: 19–30. 10.1111/j.1467-789X.2009.00613.x 19538439

[18] Crosbie EJ, Zwahlen M, Kitchener HC, Egger M and Renehan AG (2010) Body mass index, hormone replacement therapy, and endometrial cancer risk: a meta-analysis. Cancer Epidemiol Biomarkers Prev 19: 3119–3130. 10.1158/1055-9965.EPI-10-0832 21030602

[19] Discacciati A, Orsini N and Wolk A (2012) Body mass index and incidence of localized and advanced prostate cancer—a dose-response meta-analysis of prospective studies. Ann Oncol. 10.1093/annonc/mdr603 22228452

[20] Lichtman MA (2010) Obesity and the risk for a hematological malignancy: leukemia, lymphoma, or myeloma. Oncologist 15: 1083–1101. 10.1634/theoncologist.2010-0206 20930095 PMC3227901

[21] Sergentanis TN, Antoniadis AG, Gogas HJ, Antonopoulos CN, Adami HO, Ekbom A, et al (2013) Obesity and risk of malignant melanoma: a meta-analysis of cohort and case-control studies. Eur J Cancer 49: 642–657. 10.1016/j.ejca.2012.08.028 23200191

[22] Shao C, Bai LP, Qi ZY, Hui GZ and Wang Z (2014) Overweight, obesity and meningioma risk: a meta-analysis. PLoS One 9: e90167 10.1371/journal.pone.0090167 24587258 PMC3935973

[23] Higgins JPT and Green S (2011 Available from www.cochrane-handbook.org.) Cochrane Handbook for Systematic Reviews of Interventions Version 5.1.0 [updated March 2011]. The Cochrane Collaboration.

[24] Hardy RJ and Thompson SG (1998) Detecting and describing heterogeneity in meta-analysis. Stat Med 17: 841–856. 9595615 10.1002/(sici)1097-0258(19980430)17:8<841::aid-sim781>3.0.co;2-d

[25] Mittlbock M and Heinzl H (2006) A simulation study comparing properties of heterogeneity measures in meta-analyses. Stat Med 25: 4321–4333. 16991104 10.1002/sim.2692

[26] Berlin JA, Longnecker MP and Greenland S (1993) Meta-analysis of epidemiologic dose-response data. Epidemiology 4: 218–228. 8512986 10.1097/00001648-199305000-00005

[27] Il'yasova D, Hertz-Picciotto I, Peters U, Berlin JA and Poole C (2005) Choice of exposure scores for categorical regression in meta-analysis: a case study of a common problem. Cancer Causes Control 16: 383–388. 15953980 10.1007/s10552-004-5025-x

[28] Wells GA, Shea B, O'Connell D, Peterson J, Welch V, Losos M, et al. (2011) The Newcastle-Ottawa Scale (NOS) for assessing the quality of nonrandomised studies in meta-analyses. Available: http://www.ohri.ca/programs/clinical_epidemiology/oxford.asp. Accessed March 01, 2015

[29] Egger M, Davey Smith G, Schneider M and Minder C (1997) Bias in meta-analysis detected by a simple, graphical test. BMJ 315: 629–634. 9310563 10.1136/bmj.315.7109.629 PMC2127453

[30] Calle EE, Rodriguez C, Walker-Thurmond K and Thun MJ (2003) Overweight, obesity, and mortality from cancer in a prospectively studied cohort of U.S. adults. N Engl J Med 348: 1625–1638. 12711737 10.1056/NEJMoa021423

[31] Edlinger M, Strohmaier S, Jonsson H, Bjorge T, Manjer J, Borena WT, et al (2012) Blood pressure and other metabolic syndrome factors and risk of brain tumour in the large population-based Me-Can cohort study. J Hypertens 30: 290–296. 10.1097/HJH.0b013e32834e9176 22179083

[32] Holick CN, Giovannucci EL, Rosner B, Stampfer MJ and Michaud DS (2007) Prospective study of intake of fruit, vegetables, and carotenoids and the risk of adult glioma. Am J Clin Nutr 85: 877–886. 17344512 10.1093/ajcn/85.3.877

[33] Jhawar BS, Fuchs CS, Colditz GA and Stampfer MJ (2003) Sex steroid hormone exposures and risk for meningioma. J Neurosurg 99: 848–853. 14609164 10.3171/jns.2003.99.5.0848

[34] Johnson DR, Olson JE, Vierkant RA, Hammack JE, Wang AH, Folsom AR, et al (2011) Risk factors for meningioma in postmenopausal women: results from the Iowa Women's Health Study. Neuro Oncol 13: 1011–1019. 10.1093/neuonc/nor081 21750006 PMC3158016

[35] Michaud DS, Bove G, Gallo V, Schlehofer B, Tjonneland A, Olsen A, et al (2011) Anthropometric measures, physical activity, and risk of glioma and meningioma in a large prospective cohort study. Cancer Prev Res (Phila) 4: 1385–1392. 21685234 10.1158/1940-6207.CAPR-11-0014 PMC3168973

[36] Moore SC, Rajaraman P, Dubrow R, Darefsky AS, Koebnick C, Hollenbeck A, et al (2009) Height, body mass index, and physical activity in relation to glioma risk. Cancer Res 69: 8349–8355. 10.1158/0008-5472.CAN-09-1669 19808953 PMC2783605

[37] Oh SW, Yoon YS and Shin SA (2005) Effects of excess weight on cancer incidences depending on cancer sites and histologic findings among men: Korea National Health Insurance Corporation Study. J Clin Oncol 23: 4742–4754. 16034050 10.1200/JCO.2005.11.726

[38] Parr CL, Batty GD, Lam TH, Barzi F, Fang X, Ho SC, et al (2010) Body-mass index and cancer mortality in the Asia-Pacific Cohort Studies Collaboration: pooled analyses of 424,519 participants. Lancet Oncol 11: 741–752. 10.1016/S1470-2045(10)70141-8 20594911 PMC4170782

[39] Samanic C, Gridley G, Chow WH, Lubin J, Hoover RN and Fraumeni JF Jr. (2004) Obesity and cancer risk among white and black United States veterans. Cancer Causes Control 15: 35–43. 14970733 10.1023/B:CACO.0000016573.79453.ba

[40] Samanic C, Chow WH, Gridley G, Jarvholm B and Fraumeni JF Jr. (2006) Relation of body mass index to cancer risk in 362,552 Swedish men. Cancer Causes Control 17: 901–909. 16841257 10.1007/s10552-006-0023-9

[41] Wiedmann M, Brunborg C, Lindemann K, Johannesen TB, Vatten L, Helseth E, et al (2013) Body mass index and the risk of meningioma, glioma and schwannoma in a large prospective cohort study (The HUNT Study). Br J Cancer 109: 289–294. 10.1038/bjc.2013.304 23778522 PMC3708582

[42] Wolk A, Gridley G, Svensson M, Nyren O, McLaughlin JK, Fraumeni JF, et al (2001) A prospective study of obesity and cancer risk (Sweden). Cancer Causes Control 12: 13–21. 11227921 10.1023/a:1008995217664

[43] Aghi MK, Eskandar EN, Carter BS, Curry WT Jr. and Barker FG 2nd (2007) Increased prevalence of obesity and obesity-related postoperative complications in male patients with meningiomas. Neurosurgery 61: 754–760; discussion 760–751. 17986936 10.1227/01.NEU.0000298903.63635.E3

[44] Claus EB, Calvocoressi L, Bondy ML, Wrensch M, Wiemels JL and Schildkraut JM (2013) Exogenous hormone use, reproductive factors, and risk of intracranial meningioma in females. J Neurosurg 118: 649–656. 10.3171/2012.9.JNS12811 23101448 PMC3756881

[45] Custer B, Longstreth WT Jr., Phillips LE, Koepsell TD and Van Belle G (2006) Hormonal exposures and the risk of intracranial meningioma in women: a population-based case-control study. BMC Cancer 6: 152 16759391 10.1186/1471-2407-6-152 PMC1524800

[46] Lee E, Grutsch J, Persky V, Glick R, Mendes J and Davis F (2006) Association of meningioma with reproductive factors. Int J Cancer 119: 1152–1157. 16570277 10.1002/ijc.21950

[47] Little RB, Madden MH, Thompson RC, Olson JJ, Larocca RV, Pan E, et al (2013) Anthropometric factors in relation to risk of glioma. Cancer Causes Control 24: 1025–1031. 10.1007/s10552-013-0178-0 23456313 PMC3633685

[48] Cabaniols C, Giorgi R, Chinot O, Ferahta N, Spinelli V, Alla P, et al (2011) Links between private habits, psychological stress and brain cancer: a case-control pilot study in France. J Neurooncol 103: 307–316. 10.1007/s11060-010-0388-1 20835749

[49] Pan SY, Johnson KC, Ugnat AM, Wen SW and Mao Y (2004) Association of obesity and cancer risk in Canada. Am J Epidemiol 159: 259–268. 14742286 10.1093/aje/kwh041

[50] Schildkraut JM, Calvocoressi L, Wang F, Wrensch M, Bondy ML, Wiemels JL, et al (2014) Endogenous and exogenous hormone exposure and the risk of meningioma in men. J Neurosurg 120: 820–826. 10.3171/2013.12.JNS131170 24484233 PMC4386752

[51] Hill AB (1965) The Environment and Disease: Association or Causation? Proc R Soc Med 58: 295–300. 14283879 10.1177/003591576505800503 PMC1898525

[52] Renehan AG, Frystyk J and Flyvbjerg A (2006) Obesity and cancer risk: the role of the insulin-IGF axis. Trends Endocrinol Metab 17: 328–336. 16956771 10.1016/j.tem.2006.08.006

[53] Kurihara M, Tokunaga Y, Tsutsumi K, Kawaguchi T, Shigematsu K, Niwa M, et al (1989) Characterization of insulin-like growth factor I and epidermal growth factor receptors in meningioma. J Neurosurg 71: 538–544. 2552046 10.3171/jns.1989.71.4.0538

[54] Friend KE, Khandwala HM, Flyvbjerg A, Hill H, Li J and McCutcheon IE (2001) Growth hormone and insulin-like growth factor-I: effects on the growth of glioma cell lines. Growth Horm IGF Res 11: 84–91. 11472074 10.1054/ghir.2000.0183

[55] Yin D, Tamaki N, Parent AD and Zhang JH (2005) Insulin-like growth factor-I decreased etoposide-induced apoptosis in glioma cells by increasing bcl-2 expression and decreasing CPP32 activity. Neurol Res 27: 27–35. 15829155 10.1179/016164105X18151

[56] Hankinson SE, Willett WC, Manson JE, Hunter DJ, Colditz GA, Stampfer MJ, et al (1995) Alcohol, height, and adiposity in relation to estrogen and prolactin levels in postmenopausal women. J Natl Cancer Inst 87: 1297–1302. 7658481 10.1093/jnci/87.17.1297

[57] Omulecka A, Papierz W, Nawrocka-Kunecka A and Lewy-Trenda I (2006) Immunohistochemical expression of progesterone and estrogen receptors in meningiomas. Folia Neuropathol 44: 111–115. 16823693

[58] Wiemels J, Wrensch M and Claus EB (2010) Epidemiology and etiology of meningioma. J Neurooncol 99: 307–314. 10.1007/s11060-010-0386-3 20821343 PMC2945461

[59] Ismail K, Coakham HB and Walters FJ (1998) Intracranial meningioma with progesterone positive receptors presenting in late pregnancy. Eur J Anaesthesiol 15: 106–109. 9522150 10.1046/j.1365-2346.1998.00225.x

[60] Kontopantelis E, Springate DA and Reeves D (2013) A re-analysis of the Cochrane Library data: the dangers of unobserved heterogeneity in meta-analyses. PLoS One 8: e69930 10.1371/journal.pone.0069930 23922860 PMC3724681

[61] Kicinski M, Springate DA and Kontopantelis E (2015) Publication bias in meta-analyses from the Cochrane Database of Systematic Reviews. Stat Med. 10.1002/sim.6525 25988604

[62] Mullan RJ, Flynn DN, Carlberg B, Tleyjeh IM, Kamath CC, LaBella ML, et al (2009) Systematic reviewers commonly contact study authors but do so with limited rigor. J Clin Epidemiol 62: 138–142. 10.1016/j.jclinepi.2008.08.002 19013767

[63] World Cancer Research Fund / American Institute for Cancer Research (2007) Food, Nutrition, Physical Activity, and the Prevention of Cancer: a Global Perspective. Washington, DC: AICR.

