# Supplementary material for: Obesity and Risk for Brain/CNS Tumors, Gliomas and Meningiomas: A Meta-Analysis
Source: PLoS One. 2015 Sep 2;10(9):e0136974. doi: 10.1371/journal.pone.0136974 (PMC4558052; doi:10.1371/journal.pone.0136974)
Supplement: S1 File — (DOCX) [file pone.0136974.s003.docx]

**Supporting Information Methods**

*Search algorithm and eligibility of studies*

Eligible studies were sought in PubMed without any restriction of publication language; end-of-search date was June 30, 2014. The following search algorithm was used: ((("central nervous" OR CNS OR brain OR cerebral OR cerebellar) AND (tumor OR tumors OR tumour OR tumours)) OR glioma OR gliomas OR glioblastoma OR glioblastomas OR astrocytoma OR astrocytomas OR oligodendroglioma OR oligodendrogliomas OR ependymoma OR ependymomas OR meningioma OR meningiomas OR medulloblastoma OR medulloblastomas OR ganglioglioma OR gangliogliomas OR schwannoma OR schwannomas OR neurilemmoma OR neurilemmomas) AND (obesity OR "body mass" OR "body surface area" OR overweight OR obese OR BMI). The search algorithm was rather broad, so as to maximize the number of articles to be scrutinized, aiming to uncover any hidden (i.e., not apparent in the abstract) information in the full-text and tables of articles pertaining to brain cancer.

**Supporting Information Results**

*Selection of eligible studies and contact with authors*

Figure 1 presents the flow chart describing the successive steps during the selection of eligible studies. A total of 1,282 abstracts were identified and screened; 10 additional articles were retrieved through the “*snowball*” procedure [[1-10](#_ENREF_1)]. One article [[11](#_ENREF_11)] had to be excluded due to overlap with the eligible study by Aghi et al.[[12](#_ENREF_12)]; similarly, the article by Reeves et al. [[8](#_ENREF_8)] was excluded due to overlap with the larger report by Benson et al.[[13](#_ENREF_13)] on the Million Women Study. On the other hand, the Korean study by Oh et al. [[5](#_ENREF_5)] was retained in the analysis, given that its overlap with the Asian arm of the study by Parr et al. [[7](#_ENREF_7)] was minimal. Regarding the Swedish studies, both were retained in the meta-analysis, given that the study by Samanic et al. was based on the Swedish Foundation for Occupational Safety and Health of the Construction Industry Cohort [[10](#_ENREF_10)], whereas the study by Wolk et al. [[14](#_ENREF_14)] was based on the Inpatient Register of the National Board of Health and Welfare; among the latter only a minimal fraction would have been hospitalized because they were professionally active, as described in our previous meta-analysis [[15](#_ENREF_15)]. Both Swedish studies [[10](#_ENREF_10), [14](#_ENREF_14)] presented minimal overlap with the multinational studies by Edlinger et al. (Me-Can) [[16](#_ENREF_16)] and Michaud et al. (EPIC) [[17](#_ENREF_17)]; therefore all of them were included in the meta-analysis. Similarly, the Norwegian study by Wiedmann et al. [[18](#_ENREF_18)] presented minimal overlap with the multinational Me-Can study by Edlinger et al [[16](#_ENREF_16)].

Of note, the study by Moller et al. [[19](#_ENREF_19)] was not included in the meta-analysis as the classification of obesity was not based on BMI, but in most cases on the physical appearance of the subjects.

Letters were sent to the following studies due to a variety of reporting reasons. The study by Helseth et al. [[20](#_ENREF_20)] reported results with BMI as continuous variable; hence, separate categorization regarding overweight (BMI >= 25 and <30 kg/m^2^) and obese (>=30 kg/m^2^) individuals was requested, but the authors replied that the old files of the study were not available for these re-analyses. Regarding the study by Jacobs et al. [[21](#_ENREF_21)], we requested the exact BMI values corresponding to the designations "moderate obesity" and "marked obesity" stated in the manuscript, but we did not receive any reply from the authors.

With respect to the study by Schneider et al. [[22](#_ENREF_22)] we requested the underlying data regarding the statement “Results did not show a significant correlation between obesity and meningioma occurrence.” that appeared in the manuscript, but no data were provided by the authors. Concerning the articles by Preston-Martin et al. [[23](#_ENREF_23)], and Lee et al. [[24](#_ENREF_24)] where the mean body weight was compared between cases and controls, we requested results with subjects categorized in normal weight, overweight and obese, but the authors either could not provide us with data due to retirement [[23](#_ENREF_23)] or did not reply to our request [[24](#_ENREF_24)]. Similarly, no reply was received regarding the correlational study by Guo et al. [[25](#_ENREF_25)], the cohort studies by Tulinius et al. [[26](#_ENREF_26)] and Gray et al. [[27](#_ENREF_27)], where BMI was treated as a continuous variable and we requested results with subjects categorized in normal weight, overweight and obese. No reply was obtained from the authors of the studies by Hu et al. [[28](#_ENREF_28)] and Ratnasinghe et al. [[29](#_ENREF_29)], where BMI was treated as an adjustment factor; no data were available due to the oldness of the dataset by Atchison et al. [[30](#_ENREF_30)].

Finally, the letter regarding the study by Bellur et al. [[31](#_ENREF_31)] requesting the specific definition of the term “obese” in the study could not be sent, as no e-mail address could be found regarding this study published in 1983.

Letters were also sent to the eligible studies reporting overall results on brain cancer requesting separate effect estimates for gliomas and meningiomas. Unfortunately, no reply was received regarding the studies by Oh et al. [[5](#_ENREF_5)], Pan et al. [[6](#_ENREF_6)], Samanic et al. (2004)[[9](#_ENREF_9)], Samanic et al. (2006) [[10](#_ENREF_10)], Wolk et al. [[14](#_ENREF_14)], or due to retirement/decease regarding the study by Calle et al. [[2](#_ENREF_2)]. Parr et al. [[7](#_ENREF_7)] stated in their reply that stratification was not possible due to small numbers.

Additional data rendering the articles eligible were obtained regarding the study by Holick et al. [[32](#_ENREF_32)], as the authors provided us with separate relative risks for glioma regarding overweight and obese subjects.

Taken as a whole, 14 cohort studies [[2](#_ENREF_2), [5](#_ENREF_5), [7](#_ENREF_7), [9](#_ENREF_9), [10](#_ENREF_10), [13](#_ENREF_13), [14](#_ENREF_14), [16-18](#_ENREF_16), [32-35](#_ENREF_32)] (10,219 incident brain/CNS tumor cases [[2](#_ENREF_2), [5](#_ENREF_5), [7](#_ENREF_7), [9](#_ENREF_9), [10](#_ENREF_10), [13](#_ENREF_13), [14](#_ENREF_14), [16](#_ENREF_16)], 1,319 incident meningioma cases [[13](#_ENREF_13), [16-18](#_ENREF_16), [33](#_ENREF_33), [34](#_ENREF_34)], 2,418 incident glioma cases [[13](#_ENREF_13), [16-18](#_ENREF_16), [32](#_ENREF_32), [35](#_ENREF_35)], in a total cohort size of 10,143,803 subjects, Table 1) and eight case-control studies [[1](#_ENREF_1), [3](#_ENREF_3), [4](#_ENREF_4), [6](#_ENREF_6), [12](#_ENREF_12), [36-38](#_ENREF_36)] (1,009 brain/CNS tumor cases, 1,977 meningioma cases, 1,265 glioma cases and 8,316 controls, Table 2) were eligible.

**Table A.** Dose-response meta-regression analysis showing the relation between BMI (increments of 5 kg/m^2^) and risk for brain/CNS tumors, meningioma and glioma. Bold cells denote statistically significant associations.

| Gender | Method of midpoint estimation | n^§^ | Exponentiated coefficient (95%CI) | p |
| --- | --- | --- | --- | --- |
| **Overall brain/CNS** |  |  |  |  |
| Females | Berlin et al. | 10 | 1.05 (0.95-1.16) | 0.309 |
| Females | Il'yasova et al. | 9 | 1.03 (0.92-1.16) | 0.524 |
| Males | Berlin et al. | 14 | 0.99 (0.91-1.08) | 0.828 |
| Males | Il'yasova et al. | 11 | 0.97 (0.85-1.10) | 0.551 |
| Study arms not distinguishing sexes | Berlin et al. | 7 | 1.10 (0.94-1.29) | 0.164 |
| Study arms not distinguishing sexes | Il'yasova et al | 6 | 1.05 (0.89-1.24) | 0.469 |
|  |  |  |  |  |
| **Meningioma** |  |  |  |  |
| Females | Berlin et al. | 16 | **1.15 (1.03-1.28)** | 0.018 |
| Females | Il'yasova et al. | 16 | **1.17 (1.03-1.33)** | 0.023 |
| Males | Berlin et al. | 9 | 1.06 (0.81-1.39) | 0.643 |
| Males | Il'yasova et al. | 9 | 1.07 (0.79-1.47) | 0.611 |
| Study arms not distinguishing sexes | Berlin et al. | 6 | 1.13 (0.83-1.54) | 0.340 |
| Study arms not distinguishing sexes | Il'yasova et al | 6 | 1.15 (0.81-1.63) | 0.334 |
|  |  |  |  |  |
| **Glioma** |  |  |  |  |
| Females | Berlin et al. | 6 | 0.97 (0.79-1.20) | 0.728 |
| Females | Il'yasova et al. | 6 | 0.97 (0.76-1.23) | 0.728 |
| Males | Berlin et al. | 6 | 0.91 (0.57-1.47) | 0.622 |
| Males | Il'yasova et al. | 6 | 0.90 (0.52-1.55) | 0.622 |
| Study arms not distinguishing sexes | Berlin et al. | 14 | 0.95 (0.82-1.11) | 0.507 |
| Study arms not distinguishing sexes | Il'yasova et al | 13 | 0.95 (0.80-1.13) | 0.537 |

^§^number of study arms; it is occasionally smaller in the approach according to Il’yasova et al. than that per Berlin et al. due to the inherent limitations of the first algorithm in case of binary categorization of the exposure variable.

**Table B.** Evaluation of quality based on the Newcastle-Ottawa scale for the included cohort studies.

|  | **Selection** | | | | **Comparability** | | **Outcome** | | | **Total** |
| --- | --- | --- | --- | --- | --- | --- | --- | --- | --- | --- |
| **Study** | Representativeness | Selection of non-exposed | Ascertainment of exposure | Outcome not present at start | On gender | On other risk factors | Assessment of outcome | Long enough follow-up (median ≥5 years) | Adequacy (completeness) of follow-up |  |
| Benson (2008) | 1 | 1 | 0 | 1 | 1 | 1 | 1 | 1 | 1 | 8 |
| Calle (2003) | 1 | 1 | 0 | 1 | 1 | 1 | 1 | 1 | 1 | 8 |
| Edlinger (2012) | 1 | 1 | 1 | 1 | 1 | 1 | 1 | 1 | 1 | 9 |
| Holick (2007) | 0 | 1 | 0 | 1 | 1 | 1 | 1 | 1 | 1 | 7 |
| Jhawar (2003) | 0 | 1 | 0 | 1 | 1 | 1 | 0 | 1 | 1 | 6 |
| Johnson (2011) | 1 | 1 | 0 | 1 | 1 | 1 | 1 | 1 | 1 | 8 |
| Michaud (2011) | 1 | 1 | 1 | 1 | 1 | 1 | 1 | 1 | 1 | 9 |
| Moore (2009) | 1 | 1 | 0 | 1 | 1 | 1 | 1 | 1 | 1 | 8 |
| Oh (2005) | 1 | 1 | 1 | 1 | 1 | 1 | 1 | 1 | 1 | 9 |
| Parr (2010) | 1 | 1 | 0 | 1 | 1 | 1 | 0 | 0 | 0 | 5 |
| Samanic (2004) | 0 | 1 | 1 | 1 | 1 | 1 | 1 | 1 | 0 | 7 |
| Samanic (2006) | 1 | 1 | 1 | 1 | 1 | 1 | 1 | 1 | 1 | 9 |
| Wiedmann (2013) | 1 | 1 | 1 | 1 | 1 | 1 | 1 | 1 | 1 | 9 |
| Wolk (2001) | 1 | 1 | 1 | 1 | 1 | 1 | 1 | 1 | 1 | 9 |

**Table C.** Evaluation of quality based on the Newcastle-Ottawa scale for the included case-control studies.

|  | **Selection** | | | | **Comparability** | | **Exposure** | | | **Total** |
| --- | --- | --- | --- | --- | --- | --- | --- | --- | --- | --- |
| **Study** | Case definition | Representativeness of the cases | Selection of controls | Definition of controls | On gender | On other risk factors | Assessment of exposure | Same method of ascertainment for cases and controls | Non-response rate |  |
| Aghi (2007) | 1 | 1 | 0 | 0 | 1 | 0 | 1 | 1 | 0 | 5 |
| Cabaniols (2011) | 1 | 1 | 0 | 1 | 1 | 1 | 0 | 0 | 0 | 5 |
| Claus (2013) | 1 | 1 | 1 | 1 | 1 | 1 | 1 | 1 | 0 | 8 |
| Custer (2006) | 1 | 1 | 1 | 0 | 1 | 1 | 1 | 1 | 0 | 7 |
| Lee (2006) | 1 | 1 | 1 | 0 | 1 | 1 | 0 | 1 | 0 | 6 |
| Little (2013) | 1 | 1 | 1 | 0 | 1 | 1 | 0 | 1 | 0 | 6 |
| Schildkraut (2014) | 1 | 1 | 1 | 1 | 1 | 1 | 0 | 1 | 0 | 7 |

**References**

1. Cabaniols C, Giorgi R, Chinot O, Ferahta N, Spinelli V, Alla P, et al. (2011) Links between private habits, psychological stress and brain cancer: a case-control pilot study in France. J Neurooncol 103: 307-316.

2. Calle EE, Rodriguez C, Walker-Thurmond K and Thun MJ (2003) Overweight, obesity, and mortality from cancer in a prospectively studied cohort of U.S. adults. N Engl J Med 348: 1625-1638.

3. Custer B, Longstreth WT, Jr., Phillips LE, Koepsell TD and Van Belle G (2006) Hormonal exposures and the risk of intracranial meningioma in women: a population-based case-control study. BMC Cancer 6: 152.

4. Lee E, Grutsch J, Persky V, Glick R, Mendes J and Davis F (2006) Association of meningioma with reproductive factors. Int J Cancer 119: 1152-1157.

5. Oh SW, Yoon YS and Shin SA (2005) Effects of excess weight on cancer incidences depending on cancer sites and histologic findings among men: Korea National Health Insurance Corporation Study. J Clin Oncol 23: 4742-4754.

6. Pan SY, Johnson KC, Ugnat AM, Wen SW and Mao Y (2004) Association of obesity and cancer risk in Canada. Am J Epidemiol 159: 259-268.

7. Parr CL, Batty GD, Lam TH, Barzi F, Fang X, Ho SC, et al. (2010) Body-mass index and cancer mortality in the Asia-Pacific Cohort Studies Collaboration: pooled analyses of 424,519 participants. Lancet Oncol 11: 741-752.

8. Reeves GK, Pirie K, Beral V, Green J, Spencer E and Bull D (2007) Cancer incidence and mortality in relation to body mass index in the Million Women Study: cohort study. BMJ 335: 1134.

9. Samanic C, Gridley G, Chow WH, Lubin J, Hoover RN and Fraumeni JF, Jr. (2004) Obesity and cancer risk among white and black United States veterans. Cancer Causes Control 15: 35-43.

10. Samanic C, Chow WH, Gridley G, Jarvholm B and Fraumeni JF, Jr. (2006) Relation of body mass index to cancer risk in 362,552 Swedish men. Cancer Causes Control 17: 901-909.

11. Aghi MK, Eskandar EN, Carter BS, Curry WT, Jr. and Barker FG, 2nd (2007) Increased prevalence of obesity and obesity-related postoperative complications in male meningioma patients. Clin Neurosurg 54: 236-240.

12. Aghi MK, Eskandar EN, Carter BS, Curry WT, Jr. and Barker FG, 2nd (2007) Increased prevalence of obesity and obesity-related postoperative complications in male patients with meningiomas. Neurosurgery 61: 754-760; discussion 760-751.

13. Benson VS, Pirie K, Green J, Casabonne D and Beral V (2008) Lifestyle factors and primary glioma and meningioma tumours in the Million Women Study cohort. Br J Cancer 99: 185-190.

14. Wolk A, Gridley G, Svensson M, Nyren O, McLaughlin JK, Fraumeni JF, et al. (2001) A prospective study of obesity and cancer risk (Sweden). Cancer Causes Control 12: 13-21.

15. Sergentanis TN, Antoniadis AG, Gogas HJ, Antonopoulos CN, Adami HO, Ekbom A, et al. (2013) Obesity and risk of malignant melanoma: a meta-analysis of cohort and case-control studies. Eur J Cancer 49: 642-657.

16. Edlinger M, Strohmaier S, Jonsson H, Bjorge T, Manjer J, Borena WT, et al. (2012) Blood pressure and other metabolic syndrome factors and risk of brain tumour in the large population-based Me-Can cohort study. J Hypertens 30: 290-296.

17. Michaud DS, Bove G, Gallo V, Schlehofer B, Tjonneland A, Olsen A, et al. (2011) Anthropometric measures, physical activity, and risk of glioma and meningioma in a large prospective cohort study. Cancer Prev Res (Phila) 4: 1385-1392.

18. Wiedmann M, Brunborg C, Lindemann K, Johannesen TB, Vatten L, Helseth E, et al. (2013) Body mass index and the risk of meningioma, glioma and schwannoma in a large prospective cohort study (The HUNT Study). Br J Cancer 109: 289-294.

19. Moller H, Mellemgaard A, Lindvig K and Olsen JH (1994) Obesity and cancer risk: a Danish record-linkage study. Eur J Cancer 30A: 344-350.

20. Helseth A and Tretli S (1989) Pre-morbid height and weight as risk factors for development of central nervous system neoplasms. Neuroepidemiology 8: 277-282.

21. Jacobs DH, McFarlane MJ and Holmes FF (1986) Meningiomas and obesity reconsidered. Ann Neurol 20: 376.

22. Schneider B, Pulhorn H, Rohrig B and Rainov NG (2005) Predisposing conditions and risk factors for development of symptomatic meningioma in adults. Cancer Detect Prev 29: 440-447.

23. Preston-Martin S, Monroe K, Lee PJ, Bernstein L, Kelsey J, Henderson S, et al. (1995) Spinal meningiomas in women in Los Angeles County: investigation of an etiological hypothesis. Cancer Epidemiol Biomarkers Prev 4: 333-339.

24. Lee M, Wrensch M and Miike R (1997) Dietary and tobacco risk factors for adult onset glioma in the San Francisco Bay Area (California, USA). Cancer Causes Control 8: 13-24.

25. Guo WD, Linet MS, Chow WH, Li JY and Blot WJ (1994) Diet and serum markers in relation to primary brain tumor risk in China. Nutr Cancer 22: 143-150.

26. Tulinius H, Sigfusson N, Sigvaldason H, Bjarnadottir K and Tryggvadottir L (1997) Risk factors for malignant diseases: a cohort study on a population of 22,946 Icelanders. Cancer Epidemiol Biomarkers Prev 6: 863-873.

27. Gray L, Lee IM, Sesso HD and Batty GD (2012) Association of body mass index in early adulthood and middle age with future site-specific cancer mortality: the Harvard Alumni Health Study. Ann Oncol 23: 754-759.

28. Hu J, La Vecchia C, Augustin LS, Negri E, de Groh M, Morrison H, et al. (2013) Glycemic index, glycemic load and cancer risk. Ann Oncol 24: 245-251.

29. Ratnasinghe LD, Graubard BI, Kahle L, Tangrea JA, Taylor PR and Hawk E (2004) Aspirin use and mortality from cancer in a prospective cohort study. Anticancer Res 24: 3177-3184.

30. Atchison EA, Gridley G, Carreon JD, Leitzmann MF and McGlynn KA (2011) Risk of cancer in a large cohort of U.S. veterans with diabetes. Int J Cancer 128: 635-643.

31. Bellur SN, Chandra V and Anderson RJ (1983) Association of meningiomas with obesity. Ann Neurol 13: 346-347.

32. Holick CN, Giovannucci EL, Rosner B, Stampfer MJ and Michaud DS (2007) Prospective study of intake of fruit, vegetables, and carotenoids and the risk of adult glioma. Am J Clin Nutr 85: 877-886.

33. Jhawar BS, Fuchs CS, Colditz GA and Stampfer MJ (2003) Sex steroid hormone exposures and risk for meningioma. J Neurosurg 99: 848-853.

34. Johnson DR, Olson JE, Vierkant RA, Hammack JE, Wang AH, Folsom AR, et al. (2011) Risk factors for meningioma in postmenopausal women: results from the Iowa Women's Health Study. Neuro Oncol 13: 1011-1019.

35. Moore SC, Rajaraman P, Dubrow R, Darefsky AS, Koebnick C, Hollenbeck A, et al. (2009) Height, body mass index, and physical activity in relation to glioma risk. Cancer Res 69: 8349-8355.

36. Claus EB, Calvocoressi L, Bondy ML, Wrensch M, Wiemels JL and Schildkraut JM (2013) Exogenous hormone use, reproductive factors, and risk of intracranial meningioma in females. J Neurosurg 118: 649-656.

37. Little RB, Madden MH, Thompson RC, Olson JJ, Larocca RV, Pan E, et al. (2013) Anthropometric factors in relation to risk of glioma. Cancer Causes Control 24: 1025-1031.

38. Schildkraut JM, Calvocoressi L, Wang F, Wrensch M, Bondy ML, Wiemels JL, et al. (2014) Endogenous and exogenous hormone exposure and the risk of meningioma in men. J Neurosurg 120: 820-826.

**Figure A.** Forest plot describing the association between (i) overweight status, (ii) obesity and brain/CNS tumor risk among females. Apart from the overall analysis, the subanalyses on cohort (upper panels) and case-control (lower panels) studies are presented.

**(i)**

**(ii)**

**Figure B.** Forest plot describing the association between overweight status/obesity and brain/CNS tumor risk among males. Apart from the overall analysis, the subanalyses on cohort (upper panels) and case-control (lower panels) studies are presented.

**Figure C.** Forest plot describing the association between (i) overweight status, (ii) obesity and brain/CNS tumor risk among males. Apart from the overall analysis, the subanalyses on cohort (upper panels) and case-control (lower panels) studies are presented.

**(i)**

**(ii)**

**Figure D.** Forest plot describing the association between overweight status/obesity and brain/CNS tumor risk among study arms not distinguishing sexes. Apart from the overall analysis, the subanalyses on cohort (upper panels) and case-control (lower panels) studies are presented.

**Figure E.** Forest plot describing the association between (i) overweight status, (ii) obesity and brain/CNS tumor risk among study arms not distinguishing sexes. Apart from the overall analysis, the subanalyses on cohort (upper panels) and case-control (lower panels) studies are presented.

**(i)**

**(ii)**

**Figure F.** Sensitivity analysis, including studies reporting *exclusively* on brain tumors, in other words excluding the studies with collective reporting of “brain/CNS” tumors). Forest plot describing the association between overweight status/obesity and brain tumor risk among (i) females (ii) males (iii) study arms not distinguishing sexes. Apart from the overall analysis, the subanalyses on cohort (upper panels) and case-control (lower panels) studies are presented.

**(i)**

**(ii)**

**(iii)**

**Figure G.** Forest plot describing the association between (i) overweight status, (ii) obesity and meningioma risk among females. Apart from the overall analysis, the subanalyses on cohort (upper panels) and case-control (lower panels) studies are presented.

**(i)**

**(ii)**

**Figure H.** Forest plot describing the association between (i) overweight status, (ii) obesity and meningioma risk among males. Apart from the overall analysis, the subanalyses on cohort (upper panels) and case-control (lower panels) studies are presented.

**(i)**

**(ii)**

**Figure I.** Forest plot describing the association between overweight status/obesity and meningioma risk among study arms not distinguishing sexes. All study arms originated from cohort studies; the subanalyses on overweight (upper panels) and obese (lower panels) subjects are presented.

**Figure J.** Forest plot describing the association between (i) overweight status, (ii) obesity and glioma risk among females. Apart from the overall analysis, the subanalyses on cohort (upper panels) and case-control (lower panels) studies are presented.

**(i)**

**(ii)**

**Figure K.** Forest plot describing the association between overweight status/obesity and glioma risk among males. Apart from the overall analysis, the subanalyses on cohort (upper panels) and case-control (lower panels) studies are presented.

**Figure L.** Forest plot describing the association between (i) overweight status, (ii) obesity and glioma risk among males. Apart from the overall analysis, the subanalyses on cohort (upper panels) and case-control (lower panels) studies are presented.

**(i)**

**(ii)**

**Figure M.** Forest plot describing the association between overweight status/obesity and glioma risk among study arms not distinguishing sexes. Apart from the overall analysis, the subanalyses on cohort (upper panels) and case-control (lower panels) studies are presented.

**Figure N.** Forest plot describing the association between (i) overweight status, (ii) obesity and glioma risk among study arms not distinguishing sexes. Apart from the overall analysis, the subanalyses on cohort (upper panels) and case-control (lower panels) studies are presented.

**(i)**

**(ii)**

**Figure O.** Dose-response curve showing the relation between BMI and risk for meningioma (log-relative risk) in females. The circles are proportional to the inverse variance of each study and the values assigned to each BMI category were estimated according to Berlin et al.

**Figure P.** Funnel plot for the analysis examining the association between overweight status/obesity and overall brain/CNS tumor risk (i) in females, (ii) in males and (iii) in study arms not distinguishing between the two sexes.

**(i)**

**(ii)**

**(iii)**

**Figure Q.** Funnel plot for the analysis examining the association between overweight status/obesity and meningioma risk (i) in females, (ii) in males and (iii) in study arms not distinguishing between the two sexes.

**(i)**

**(ii)**

**(iii)**

**Figure R.** Funnel plot for the analysis examining the association between overweight status/obesity and glioma risk (i) in females, (ii) in males and (iii) in study arms not distinguishing between the two sexes.

**(i)**

**(ii)**

**(iii)**
